# First Polycipivirus and Unmapped RNA Virus Diversity in the Yellow Crazy Ant, *Anoplolepis gracilipes*

**DOI:** 10.3390/v14102161

**Published:** 2022-09-30

**Authors:** Chih-Chi Lee, Hung-Wei Hsu, Chun-Yi Lin, Nicolas Gustafson, Kenji Matsuura, Chow-Yang Lee, Chin-Cheng Scotty Yang

**Affiliations:** 1 Laboratory of Insect Ecology, Graduate School of Agriculture, Kyoto University, Kyoto 6068502, Japan; 2 Research Institute for Sustainable Humanosphere, Kyoto University, Kyoto 6110011, Japan; 3 Department of Evolutionary and Environmental Biology, Institute of Evolution, University of Haifa, Haifa 3498838, Israel; 4 Institute of Biomedical Sciences, Academia Sinica, Taipei 115, Taiwan; 5 Citrus Research and Education Center, University of Florida, Lake Alfred, FL 33850, USA; 6 Department of Biochemistry, Virginia Polytechnic Institute and State University, Blacksburg, VA 24061, USA; 7 Department of Entomology, University of California, 900 University Avenue, Riverside, CA 92521, USA; 8 Department of Entomology, Virginia Polytechnic Institute and State University, Blacksburg, VA 24061, USA

**Keywords:** invasive ant, insect virus, *polycipiviridae*, *Sopolycivirus*, picornavirus, virome, RNA virus

## Abstract

The yellow crazy ant, *Anoplolepis gracilipes* is a widespread invasive ant that poses significant threats to local biodiversity. Yet, compared to other global invasive ant species such as the red imported fire ant (*Solenopsis invicta*) or the Argentine ant (*Linepithema humile*), little is known about the diversity of RNA viruses in the yellow crazy ant. In the current study, we generated a transcriptomic database for *A*. *gracilipes* using a high throughput sequencing approach to identify new RNA viruses and characterize their genomes. Four virus species assigned to *Dicistroviridae*, two to *Iflaviridae*, one to *Polycipiviridae*, and two unclassified *Riboviria* viruses were identified. Detailed genomic characterization was carried out on the polycipivirus and revealed that this virus comprises 11,644 nucleotides with six open reading frames. Phylogenetic analysis and pairwise amino acid identity comparison classified this virus into the genus *Sopolycivirus* under *Polycipiviridae*, which is tentatively named “*Anoplolepis gracilipes* virus 3 (AgrV-3)”. Evolutionary analysis showed that AgrV-3 possesses a high level of genetic diversity and elevated mutation rate, combined with the common presence of multiple viral strains within single worker individuals, suggesting AgrV-3 likely evolves following the quasispecies model. A subsequent field survey placed the viral pathogen “hotspot” of *A. gracilipes* in the Southeast Asian region, a pattern consistent with the region being recognized as part of the ant’s native range. Lastly, infection of multiple virus species seems prevalent across field colonies and may have been linked to the ant’s social organization.

## 1. Introduction

Several life history traits common to invasive ants, such as polygyne (i.e., multiple reproductive queens in an ant colony) and supercolony (i.e., ants from physically separated colonies show reduced aggression towards each other), have been believed to contribute to their ecological dominance in their introduced ranges [1,2,3]. However, such life history traits have been linked to the increased risks of pathogen exposure/transmission and, thus, high pathogen diversity/load associated with colony-level pathogen buffering mechanism [4,5,6]. For example, colonies of polygyne social form generally harbor a higher viral load than monogyne (i.e., single reproductive queen in an ant colony) in the red imported fire ant (*Solenopsis invicta*) [7,8,9]. Infection with multiple viruses is also common in the invasive Argentine ant (*Linepithema humile*), a polygynous, supercolony-forming ant species [10,11,12].

The yellow crazy ant, *Anoplolepis gracilipes*, is a globally distributed invasive ant; yet, the native range of this ant species remains debatable as multiple datasets failed to reach consensus [13]. However, new lines of evidence have been recently accumulated to support the Southeast Asian origin of this ant [14,15,16]. High enemy/pathogen diversities and loads may indicate an invasive species’ native range as invasion likely filters out certain enemies/pathogens due to invading propagules representing only a small proportion of the native or source population [17]. While a few pathogens have been reported to be associated with this ant, only two dicistroviruses (with well-characterized genomes) [18] and three dicistrovirus-like sequences were identified [19]. Compared to other globally invasive ants, such as the Argentine ant and red imported fire ant [10,20], the number of virus species reported in *A*. *gracilipes* is relatively low. More efforts are therefore required to better understand the viral diversity of this invasive ant species. 

This study aims to explore the virus diversity in this ant species with a focus on RNA viruses and their genetic diversities and evolution. We used high throughput sequencing to generate a transcriptomic database for *A*. *gracilipes*, which was then used to identify and characterize new virus genomes and other virus-like sequences. We also surveyed the presence of these viruses across the ant’s geographical range and examined if colonies collected from Southeast Asian regions harbor higher levels of virus diversity. Lastly, genetic analyses were carried out to assess the genetic diversity and selection signals for one of the discovered viruses (*Anoplolepis gracilipes* virus 3, See Results) associated with the yellow crazy ant, which is expected to facilitate the understanding of evolutionary dynamics of virus-ant interactions.

## 2. Materials and Methods

### 2.1. Sample Collection, RNA Purification, and Sequencing

Two *A*. *gracilipes* colonies were collected from the roadside of semi-natural habitat, with one from Penang, Malaysia (5°21′32.4″ N, 100°18′09.0″ E) and the other from Okinawa, Japan (26°40′19.2″ N, 128°00′41.0″ E). Penang is considered part of the putative native range of *A*. *gracilipes*, while Okinawa is located in the ant’s known invasive range [13]. A single worker individual from each of the two colonies was randomly selected to establish an RNA-pool for RNA sequencing. The entire worker ant was soaked and homogenized with a pestle in TRIzol™ RNA Extraction Reagent (Invitrogen, Carlsbad, CA, USA), and the standard TRIzol RNA extraction protocol was followed. The RNA quality and quantity were measured using a NanoDrop spectrophotometer and then mixed at a 1:1 ratio based on the total RNA concentration of each sample. The pooled RNA was submitted to Eurofins Genomics (Tokyo, Japan) for sequencing after RNA purification using the polyA selection method. The sequencing library was constructed with the TruSeq^®^ Stranded mRNA Library Prep (Illumina, San Diego, CA, USA) with an average 200 bp library insert size. The library was sequenced in the pair-end mode (2 × 100 bp) using the Illumina HiSeq 4000 platform.

### 2.2. De Novo Assembly and Virus Characterization

The pair-end reads obtained from Illumina sequencing were trimmed to remove adaptors and low-quality sequences (q < 28; base call accuracy < 99.8%) using Trimmomatic v0.36 [21]. The de novo assembly of transcripts was generated using Trinity v2.8.4 [22]. The assembly was compared against the NCBI GenBank non-redundant (nr) protein database using BLASTx [23] implemented in Diamond v0.9.21 [24] with an e-value < 1 × 10^−40^. The virus-like transcripts were identified using MEGAN Community Edition v6.18.8 [25]. The open reading frames (ORFs) of putative viral-like transcripts were identified using NCBI ORF finder [26], and conserved domains were discriminated using HHpred [27] against Pfam [28] and PDB [29] databases. The pair-end reads were mapped onto Trinity contigs using Bowtiew2 v2.4.1 [30], and the average read depth per nucleotide was then calculated using SAMtools v1.10 [31].

The potential viral ORFs were subjected to a preliminary taxonomic inference using BLASTp [23] that compares the identified virus-like sequence ORFs against the NCBI non-redundant protein database (nr). Phylogenetic analysis was performed on these potential viral ORFs with top hits against the clustered nr database (nr_clustered) by BLASTp [23], followed by the construction of fast minimum evolution phylogenetic tree with a maximum sequence difference of 0.85 in Grishin distance using Blast Tree View and visualized in iTOL v5 [32]. 

### 2.3. Virus Verification and Field Virus Prevalence

To verify the occurrence of the identified viruses in the two original *A*. *gracilipes* colonies, ten worker ants from each of the two colonies were randomly selected for RNA purification. To investigate the prevalence of these viruses in the field, additional 107 yellow crazy ant colonies were collected from Malaysia (n = 35), Indonesia (n = 4), Taiwan (n = 40), Okinawa (n = 18), and Hawaii (n = 10). Malaysia and Indonesia have been considered part of the putative native range of *A*. *gracilipes*, while Taiwan, Okinawa, and Hawaii are located in the ant’s known invasive range [13]. The total RNA was bulk extracted from ten worker ants from each of the 107 colonies following the same procedure mentioned above. 

The virus-specific primers were designed using Primer3 [33]. First strand cDNA was synthesized with oligo dT primer using PrimeScript^TM^ 1st strand cDNA Synthesis kit (Takara Bio Inc., Shiga, Japan) under conditions as follows: 30 °C for 10 min., 42 °C for 60 min., followed by a final inactivation step at 95 °C for 5 min. The cDNA then served as a template for PCR reaction amplifying the partial sequence of RNA-dependent RNA polymerase (RdRp) or Capsid protein region using respective virus-specific primer sets (Table S1). The PCR cycling profile was as follows: 94 °C for 3 min, 35 cycles of 94 °C for 30 s, 60 °C for 30 s, and 72 °C for 60 s, with a final extension step of 72 °C for 5 min. Positive and negative controls were included in each batch of PCR reactions. A separate PCR targeting a host gene, *EF1-α*, was also carried out as an internal control to account for false-negative reactions due to PCR/RNA extraction failure, using primers Agr_EF1a.for (5′-CCTGGGTGTTGGACAAACTT-3′) and Agr_EF1a.rev (5′GCTCCTTCACCGAGATGTTC-3′) (Table S1).

### 2.4. Phylogenetic and Evolutionary Analysis

Taxonomic placement of the identified virus was performed using a phylogenetic approach. We collected sequences of multiple polycipiviruses containing full non-structural polyprotein (ORF5) sequences from GenBank. A total of 28 polycipiviruses, including three chipolyciviruses, one hupolycivirus, 14 *Sopolyciviruses*, and additional ten unclassified polycipiviruses, were used to reconstruct the phylogeny with three iflaviruses as outgroup. Amino acid sequences of the entire non-structural polyprotein (ORF5) were aligned using MUSCLE implemented in MEGA 7 [34]. The best evolutionary model was calculated by ModelTest-NG v0.1.6 [35], and the LT+I+G4 model was determined as the best fit according to the Akaike information criterion and Bayesian information criterion. The phylogenetic tree based on the maximum likelihood method was reconstructed using RAxML-NG v0.9.0 [36] with 1,000 bootstrap replicates. The tree convergence was verified with the weighted Robinson-Foulds (WRF) distance < 3% as the cutoff. The Bayesian inference method was implemented in MrBayes v3.2.7 [37] with 1,000,000 generations (sampling every 500 generations, with 25% burn-in). VT+I+G4 was identified as the best-fit substitution model for MrBayes [37], as estimated using ModelTest-NG v0.1.6 [35]. The convergence diagnostic was assessed by a potential scale reduction factor (PSRF) = 1 and the estimated sample size (ESS) > 100. The convergent phylogenetic tree was then visualized in iTOL v5 [32] and rooted at the iflaviruses. To confirm whether the identified polycipivirus sequences were novel viruses, a pairwise comparison of the amino acid identities of the entire ORF5 (non-structural polyprotein) against 28 polycipiviruses obtained from GenBank was performed using BLASTp [23].

To examine whether our Trinity contigs originate from the same or different host individuals (i.e., either from Malaysia or Okinawa), we reconstructed the virus phylogeny based on 13 partial sequences of RdRp region from 11 field-collected *A*. *gracilipes* colonies (Malaysia n = 5, Taiwan n = 2, Okinawa n = 2, and Indonesia n = 2). RNA of a single randomly-selected worker from each colony was purified, and cDNA was synthesized following the same procedure mentioned above. The synthesized cDNA was then used as a template for PCR that amplifies partial sequences of RdRp region, using the following primers, AgrV3_RdRp.for (3′- CAAAGTGAATGACCCCGAGT-5′) and AgrV3_RdRp.rev (3′-ATGCGAGAAATCGTGTTTCC-5′) primers (Table S1). PCR products were cloned using an NEB ^®^ PCR cloning kit (New England BioLabs, Ipswich, MA, USA), and up to ten clones were subjected to Sanger sequencing. Two individuals (one from Malaysia and one from Indonesia) were found to have two different haplotypes among the ten sequenced clones, whereas only a single haplotype was recovered from the sequenced clones for the rest of the individuals. Six Trinity assembled transcripts (isoforms DN93_c0_g1_i2, i4, i6-i9; Supplementary Data S1) that contained RdRp region, two *Sopolyciviruses*, Lasius neglectus virus 1 (GenBank: ASK12200.1) and Myrmica scabrinodis virus 1 (GenBank: ASK12206.1), were also included in the reconstruction of the phylogenetic tree. These sequences were aligned using MUSCLE implemented in MEGA 7 [34]. The best evolutionary model was estimated by jModelTest v2.2.10 [38], and the GTR+I+G model was determined as the best substitution model by both Akaike information criterion and Bayesian information criterion. The phylogenetic tree based on the maximum likelihood method was reconstructed by MEGA 7 [34] with 1000 bootstrap replicates and the Bayesian inference method using MrBayes v3.2.7 [37] with 1,000,000 generations (sampling every 500 generations, with 25% burn-in).

To examine whether quasispecies exist in the identified polycipivirus and characterize the selection pattern, we estimated Tajima’s D on the RdRp region from 13 sequences of field-collected colonies using MEGA 7 [34]. The nonsynonymous substitutions (dN) and synonymous substitutions (dS) were estimated using yn00 implemented in PAML-X [39].

## 3. Results

### 3.1. Characterization of the Virus Genome

The results of BLASTx and MEGAN analysis identified 34 virus-like transcripts with e-value < 1 × 10^−40^ out of a total of 70,145 trinity transcripts assembled by Trinity. The preliminary taxonomic inference with BLASTp and fast minimum evolution phylogenetic tree placed these virus-like transcripts into one of four virus families, including 11 *Dicistroviridae*, 3 *Iflaviridae*, 18 *Polycipiviridae*, and 2 unclassified *Riboviria* (Table 1, Figure S1, Supplementary Data S1). These virus-like contigs possessed the mean read support per nucleotide (read depth) ranging from 3.52× to 50,371.26×. The amino acid identity of ORF in each virus-like sequence to the best hit in the NCBI database ranged from 41.94% to 75.22%. None of our virus-like transcripts presented over 90% amino acid identity to any existing viral sequences, indicating that each sequence likely represented a novel virus species. We described more details of these potential viruses in the following paragraphs.

### 3.2. Virus Identification

#### 3.2.1. *Polycipivirus*

**Figure 1:**
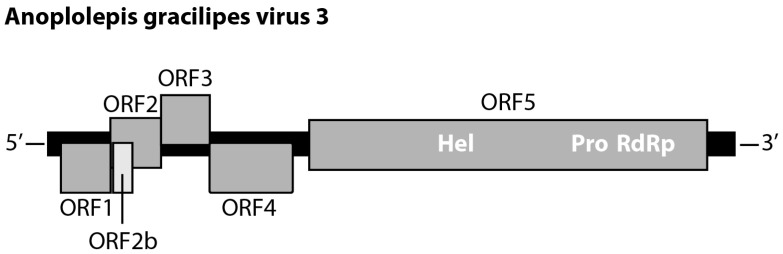
Genome structure of *Anoplolepis gracilipes* virus 3 (GenBank: MW078933). ORFs are represented by grey rectangles with vertical offsets indicating reading frames (−1, 0, +1) relative to the Hel-Pro-RdRp ORF (ORF5). Hel: RNA helicase, Pro: protease, RdRp: RNA-dependent RNA polymerase.

Two of 18 *Polycipiviridae*-like transcripts were identical in length (11,644bp, excluding poly-A tail). For the other 16 transcripts, pairwise comparisons of any two transcripts revealed >80% nucleotide identity. These two transcripts contained the same genomic feature that possesses five main ORFs: ORF1 (nucleotide (nt) 218–1063; 281 amino acids (aa)), ORF2 (nt 1060–1920; 286 aa), ORF3 (nt 1920–2747; 275 aa), ORF4 (nt 2744–4156; 470 aa), and ORF5 (nt 4438–11,196; 2252 aa). Moreover, a short ORF2b (nt 1106–1432; 108 aa) was located within the ORF2 (Figure 1). The alignment of the two transcripts showed that there are 27 synonymous mutations in ORF5. The amino acid sequences of the six ORFs were identical between the two transcripts. The results of the HHpred search predicted ORF1, 2, 3, and ORF4 are putative Capsid proteins, and ORF2b was a putative protein associated with protein translocation. The ORF5 contained RNA helicase (nt 6613–6922), protease (nt 8881–9505), and RNA-dependent RNA polymerase (nt 9649–11,158). The top hit of ORF5 compared against the NCBI nr database was Lasius neglectus virus 1 (GenBank: ASK12200.1), with 58% amino acid identity (1318/2282 aa). The pairwise comparison of the amino acid sequence of ORF5 against the other 27 current known polycipiviruses indicated that Lasius neglectus virus 1 shared the highest pairwise identity (58%) with the focal transcript. Given the fact that no current known viruses shared more than 90% amino acid sequence identity of non-structure protein (ORF) to this transcript, we thus proposed a new virus and tentatively named it “*Anoplolepis gracilipes* virus 3 (AgrV-3)” (GenBank: MW078933).

#### 3.2.2. *Dicistrovirus*-like Fragments

**Figure 2:**
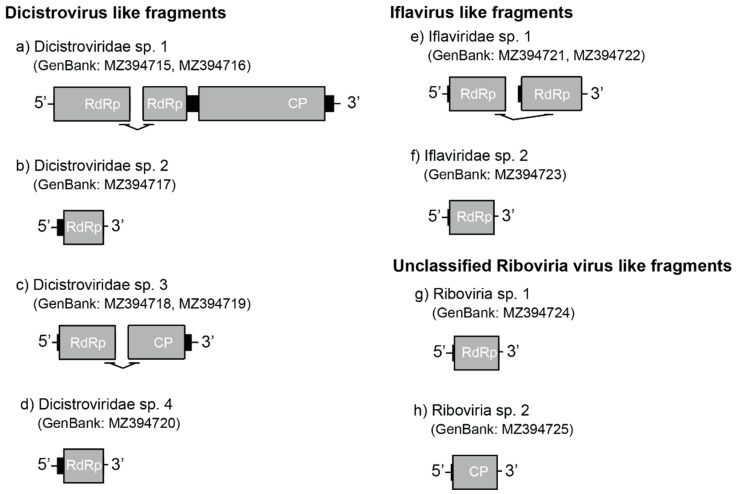
The conserved domain of virus-like fragments in the *A*. *gracilipes* transcriptome. Grey rectangles represent ORFs. RdRp: RNA-dependent RNA polymerase, CP: capsid protein.

In addition to five transcripts that belonged to one of the two previously described dicistroviruses in this ant [18], we found six partial dicistrovirus-like sequences assigned to four unique viruses. The first two dicistrovirus-like sequences (YCA-associated putative *Dicistroviridae* sp. 1; GenBank: MZ394715, MZ394716) comprised two contigs (952 nt and 6015 nt; Figure 2a) that were bridged by eight paired-reads. The result of HHpred showed the 5′ end contig presented a partial putative RdRp sequence. The following long contig contained the partial sequence of a putative RdRp and a putative structural polyprotein (capsid protein; CP). The third dicistrovirus-like sequence (YCA-associated putative *Dicistroviridae* sp. 2; GenBank: MZ394717) was 413 nt and possessed a partial putative RdRp sequence (Figure 2b). The fourth and fifth sequences (YCA-associated putative *Dicistroviridae* sp. 3; GenBank: MZ394718, MZ394719) were derived from two contigs (744 nt and 1226 nt), respectively, and were joined by four pairs of reads. The conserved domain in the 5’ end contig of YCA-associated putative *Dicistroviridae* sp. 3 was a partial putative RdRp region. A putative capsid protein was identified in the following contig (Figure 2c). The last dicistrovirus-like sequence (YCA-associated putative *Dicistroviridae* sp. 4; GenBank: MZ394720) was 732 nt, containing a partial putative RdRp region (Figure 2d). The protein identity between four dicistroviruses ranged from 22.22% to 45.46% if an overlap region exists (Table S2).

Phylogenetic examination indicated that YCA-associated putative dicistrovirus 1, 2, and 4 are closely related to Wuhan arthropod virus 2 (GenBank: NC_033437.1; Figure S1b,c,e), sharing 60.25% amino acid identity (5′ end contig of *Dicistroviridae* sp. 1; GenBank: MZ394715), 70.72% (3′ end contig of *Dicistroviridae* sp. 1; GenBank: MZ394716), 66.42% (*Dicistroviridae* sp. 2), and 75.22% (*Dicistroviridae* sp. 4) with Wuhan arthropod virus 2, respectively. The YCA-associated putative dicistrovirus 3 was closely related to Solenopsis invicta virus 1A (GenBank: AY831776.1; Figure S1d) with 51.49% of amino acid identity. 

#### 3.2.3. *Iflavirus*-like Fragments

The YCA-associated putative *Iflaviridae* sp. 1 (GenBank: MZ394721, MZ394722) contained two contigs (471 nt and 440 nt, respectively) that were connected by two pairs of cross contig reads. Both contigs were the partial region of a putative RdRp (Figure 2e; Figure S1f). The top and well classified hit of BLASTp was Moku virus (GenBank: YP_009305421.1, 66.61% protein identity) for the 5’ end contig and Vespula vulgaris Moku-like virus (GenBank: QZZ63303.1, 70.63% protein identity) for the following contig. The YCA-associated putative *Iflaviridae* sp. 2 (GenBank: MZ394723) was 2006 nt, which contained a partial region of a putative RdRp (Figure 2f). YCA-associated putative *Iflaviridae* sp. 2 was most genetically related to Vespa velutina Moku virus (GenBank: ATY36108.1, 66.96% protein identity). The protein identity between YCA-associated putative *Iflaviridae* sp. 1 and sp. 2 were 79.49% (5’ end contig) and 81.94% (3’ end contig), respectively. The phylogeny of *Iflaviridae* sp. 1 and 2 were within the branch of *Iflaviridae* (Figure S1f).

#### 3.2.4. Unclassified *Riboviria*-like Fragments 

The YCA-associated putative *Riboviria* sp. 1 (GenBank: MZ394724) contained a partial putative 560 nt RdRp region (Figure 2g). The BLASTp top hit was HVAC-associated RNA virus 1 (GenBank: AVD69111.1), sharing 48.82% of amino acid identity. The YCA-associated putative *Riboviria* sp. 2 (GenBank: MZ394725) was 1636 nt and contained a putative partial Capsid protein region (Figure 2h). The YCA-associated putative *Riboviria* sp. 2 was most genetically related to unclassified *Riboviria* sp. (GenBank: QKN88918.1) with 55.43% identity. No overlapping region was found in the two *Riboviria* sp. contigs. The fast minimum evolution phylogenetic tree supported two contigs both were picornaviruses (Figure S1g,h).

### 3.3. Phylogenetic Analysis and Polycipivirus Classification

**Figure 3:**
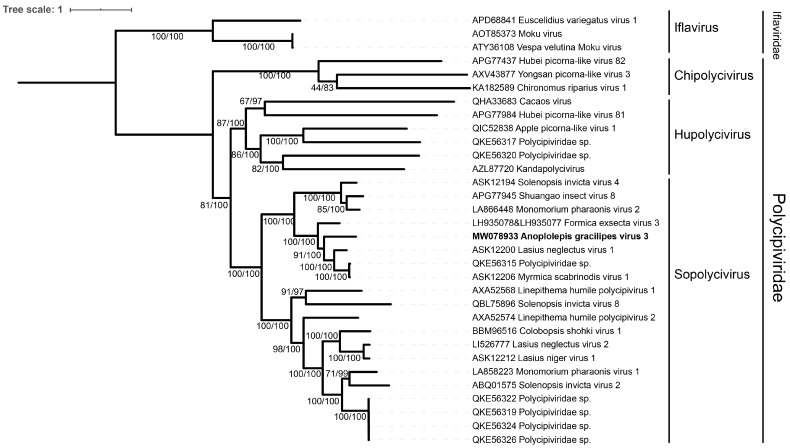
Phylogeny of *Anoplolepis gracilipes* virus 3, other 28 *Polycipiviridae* and 3 *Iflaviridae* viruses based on the ORF5. The phylogenetic tree was constructed by the maximum likelihood and Bayesian inference methods. The numbers at each node indicate the bootstrap supporting value and the posterior probability, respectively. The phylogenetic tree was rooted using three *Iflaviridae* viruses. The GenBank accessions are followed by virus species names.

The Bayesian inference method produced a tree topologically identical to the maximum likelihood tree, showing AgrV-3 is most closely related to viruses in the genus of *Sopolycivirus* (Figure 3). Our phylogenetic analyses also placed those previously published yet unassigned *Polycipiviridae* viruses into one of three genera: the genus of *Chipolycivirus*: Yongsan picorna-like virus 3 (host: mosquito, *Ades vexans nipponii*) [40], the genus of *Hupolycivirus*: Kandapolycivirus (host: bat, *Pteropus lylei*) [41], and Apple picorna-like virus 1 (host: apple leaf, *Malus domestica*, Honeycrisp G.935) [42].

### 3.4. Field Prevalence and Evolutionary Analysis

RT-PCR verification of viral sequences from the original two colonies indicated that most of the viruses were only found in the Malaysian colony. We also conducted a field survey for the presence of all viruses and found that most of the viruses were only detected in their putative native ranges (Malaysia and Indonesia) with low infection rates but completely absent in the ant’s known invasive range (Taiwan, Okinawa, and Hawaii; Table S3). One exception exists: AgrV-3 was the only virus species found to persist in most of the surveyed ant samples across regions with varying prevalence levels. High infection rates of AgrV-3 (proportion of infected colonies) were found in Malaysia (60%; 21/35) and Indonesia (50%; 2/4), followed by Taiwan (35%; 14/40) and Okinawa (11%; 2/18). No colonies were infected with AgrV-3 in Hawaii (0%, n = 10).

**Figure 4:**
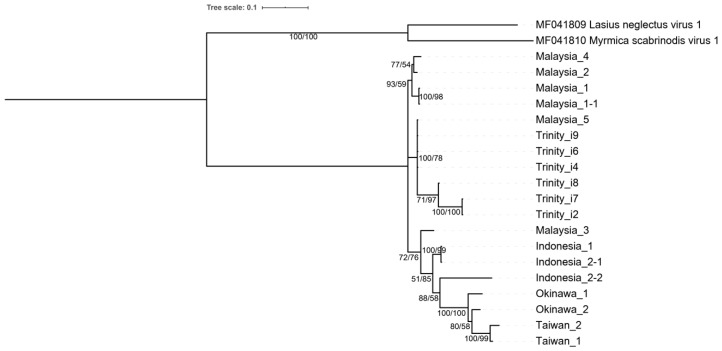
Phylogeny of *Anoplolepis gracilipes* virus 3 based on RdRp region. A total of 13 sequences obtained from field-collected colonies and six Trinity transcripts were included. The phylogenetic tree was constructed using both the maximum likelihood and Bayesian inference methods. The numbers at each node indicate the bootstrap supporting value and the posterior probability, respectively. The tree was rooted at the Lasius neglectus virus 1 and Myrmica scabrinodis virus 1 clade.

Phylogenetic analysis based on the RdRp region (partial ORF5) of six *Polycipiviridae*-like transcripts and 13 AgrV-3 sequences from 11 field ant colonies showed all *Polycipiviridae*-like transcripts from our assembly (DN93_c0_g1_i2, i4, i6-i9; Supplementary Data S1) were more closely related to the viral sequences recovered from *A*. *gracilipes* in Malaysia than those from Okinawa (Figure 4). This is consistent with the results of RT-PCR verification, where AgrV-3 was only recovered from the Malaysian colony. Interestingly, two viral sequences from the same ant colony in Indonesia (Indonesia_2-1 & Indonesia_2-2) were different strains, sharing 91% nucleotide identity (464/510). The overall Tajima’s D was 0.3969, with the genetic diversity (pi) of 0.0796. The average nonsynonymous substitution rate (dN) was 0.0115, whereas the synonymous substitution rate (dS) was 0.4988. The dN/dS was 0.023.

## 4. Discussion

While most viruses discovered from ants are single-stranded RNA viruses belonging to the order *Picornavirales* especially in the families of *Dicistroviridae*, *Iflaviridae*, and *Polycipiviridae*, other RNA viruses (e.g., *Nyamiviridae*, *Rhabdoviridae*, *Nodaviridae*, *Solinviviridae*, and *Totiviridae*) and dsDNA viruses (e.g., *Baculoviridae*, *Myoviridae*, *Parvoviridae*, *Podoviridae*, *Poxviridae*, and *Siphoviridae*) have also been reported in ants [43]. Methods to discover novel viruses in ants included PCR-based cloning and sequencing or expression library sequencing [44,45], and, more recently, high throughput sequencing techniques (e.g., Illumina platforms) such as metatranscriptomics (mRNA/RNA-seq) [7,10,11,12,18,19,20,40,41,42,43]. Metagenomics approach is particularly powerful as it has facilitated uncovering RNA viruses [43], ssDNA viruses [46], and dsDNA [47] in ants and across other taxa. The current study reports a new viral genome (*Anoplolepis gracilipes* virus 3, AgrV-3) as well as several unmapped (partially characterized) viruses in the yellow crazy ant through a transcriptomic approach. *Anoplolepis gracilipes* virus 3 represents the first *Polycipiviridae* virus and also the third complete virus genome that has been discovered in this invasive ant species.

The *Polycipiviridae* is a relatively new virus family (2017) of Picornavirus [48,49] with a genome composed of a positive-sense liner RNA of approximately 10–12 kb that includes four 5’-proximal open reading frames (ORFs) and one long 3’ ORF (ORF5). Three genera are currently reported in *Polycipiviridae*, including *Sopolycivirus*, *Hupolycivirus*, and *Chipolycivirus* [48,49]. Our analyses, including the genomic structure (ORF number, putative functions, and orientation), pairwise amino acid identity, and phylogeny, support the placement of this virus in the genus of *Sopolycivirus* under the family *Polycipiviridae*. Based on the species demarcation criteria established by ICTV [49], no currently known viruses in *Polycipiviridae* [40,41,42,48,49,50,51,52,53] present >90% amino acid identity to ORF5 (non-structural polyprotein) of this virus. We thus propose a new virus tentatively named “*Anoplolepis gracilipes* virus 3” (GenBank: MW078933).

Our analysis identified six ORFs in *Anoplolepis gracilipes* virus 3 (hereafter: AgrV-3) with putative ORF functions and orientation (Figure 1) resembling other polycipiviruses [48,49]. The phylogenetic relationships of the entire AgrV-3 ORF5 with other 28 polycipiviruses and 3 iflaviruses by both maximum likelihood and Bayesian inference methods provide robust evidence in support of AgrV-3 being a member of *Sopolycivirus* (Figure 3). Viruses in this family are positive-sense single-stranded RNA viruses that infect arthropods and were mainly discovered in ants [48,49,50,51,52]. Interestingly, polycipiviruses in the genus of *Hupolycivirus* appear to have diverse host utility as Apple picorna-like virus 1 was detected in the leaf tissue of apple trees [42], while Kandapolycivirus was found to infect bats [41]. Solenopsis invicta virus 2 (SINV-2) infecting *Solenopsis* fire ants is a member of *Sopolycivirus* [48]. Although little is known about the pathogenesis of this virus group of viruses in ants, a previous study indicates that SINV-2 could significantly alter fire ant fitness through reduction of queen reproduction, ultimately impeding success of colony founding [54]. We are currently generating necessary data to establish potential fitness costs associated with AgrV-3 infection in *A*. *gracilipes*.

Our de novo Trinity assembly reconstructed two full (or near full) length (including 5’-end untranslated region, six ORFs, and polyA tail) and 16 partial contigs that possess high nucleotide identity (>80%) against each of the two full-length contigs. As our assembly derived from a pooled RNA (a worker individual from Malaysia and Okinawa), we constructed a phylogeny tree that includes six RdRp containing transcripts (DN93_c0_g1_i2, i4, i6-i9; Supplementary Data S1) to those sequences obtained from the field samples by Sanger sequencing to investigate the origin of these transcripts (i.e., from either Malaysia or Okinawa). The finding that all transcripts from our Trinity assembly are more closely related to those Malaysian origin isolates indicates that AgrV-3 is most likely recovered from the ant from Malaysia rather than from Okinawa. Further, the result also suggests the possibility of multiple virus strains co-existing in a single ant individual since the Okinawa ant does not appear to contribute to any of these transcripts. This possibility is well supported by another individual from Indonesia, where two different AgrV-3 strains (Indonesia_2-1 & Indonesia_2-2) were detected. The quasispecies concept is one of the potential explanations for multiple viral strains in a single host. The rapid and error-prone replication of an RNA virus tends to have an elevated mutation rate and thus readily forms a “mutant cloud” of variants [55].

The presence of diverse variants has been thought to contribute to rapid adaptations of viruses to dynamic environments in the hosts, such as escaping from the host immune or even transmission to a novel host [55]. Multiple AgrV-3 strains infecting a single *A*. gracilipes individual or an ant colony is most likely a consequence of error-prone viral replication. The positive value of Tajima’s D in our analysis indicates the observed genetic diversity is greater than the expected genetic diversity, hence supporting the rapid evolution and elevated mutation rate in this virus. Furthermore, the analysis of substitution rate at silent sites (dS) is 0.4988, a pattern that is comparable to another ant-infecting *Sopolycivirus* (Linepithema humile polycipivirus 1, LhuPcV1; host: Argentine ant) [10]. Interestingly, in the same study [10], LhuPcV1 was shown to possess higher dS than two other single-stranded RNA viruses, Kashmir bee virus (*Dicistroviridae*) and Linepithema humile C-virus 1 (unclassified *Riboviria*), in Argentine ants [10], providing further evidence in support of high genetic diversity and likely high level of host adaptability in polycipiviruses. The observed low dN/dS ratio for AgrV-3 in *A. gracilipes* suggests that a purifying selection is potentially acting on the RdRp region for optimizing the virus’ fitness in the yellow crazy ant. The similarity in the genetic diversity and evolutionary pattern between the two polycipiviruses raises an interesting question of whether the presence of diverse variants is a common characteristic in *Polycipivirus* and highlights the need of further research on the evolutionary dynamics of viruses in this newly established virus family. 

In addition to AgrV-3, our dataset also reports several additional contigs containing six viruses in *Dicistroviridae* (including two previously published triatoviruses, AgrV-1 and AgrV-2 [18]), two in *Iflaviridae*, and two unclassified *Riboviria*. Moreover, most of these viruses have been confirmed to predominately originate from the original Malaysian *A*. *gracilipes* worker, indicating this particular worker that has been sequenced harbors multiple viruses. We suspect the yellow crazy ant’s polygynous social organization may account for the observed virus diversity. For example, infection of multiple viruses seems more common in ant species expressing the polygyne social trait compared to monogyne [7,8,9], and this is most likely due to the near absence of nest boundary, frequent interactions, and/or the free exchange of individuals between nests that encourage viral transmission among nests once a novel virus is introduced in the population [1,5,7]. The polygynous trait also potentially leads the population to be a reservoir of a broad pathogen spectrum through improving colony disease tolerance due to colony-level pathogen buffering mechanisms [4,5]. 

Recently, evidence has emerged to support the Southeast Asian origin of the yellow crazy ant [13,14,15,16,56]. These data include localized supercolony structure, independent colony founding, and the presence of rare, divergent mtDNA haplotype in *A*. *gracilipes* across southeast Asia. Unlike these data deriving from the ant host, our study provided evidence from the perspective of the viral pathogens infecting the host. The pattern of virus species diversities placed the pathogen “hotspot” of *A. gracilipes* in this particular geographical region. This result, together with the finding that the RdRp sequences recovered from Malaysia are ancestral to those recovered from the known introduced ranges (Figure 4), favors the Southeast Asian origin of the yellow crazy ant.

Pathogens are often found to be co-introduced with their hosts via human-mediated transportation network [57]. Arrival of these co-introduced viral pathogens may threaten native ant/arthropod communities in the invasive range due to virus spillover [58]. Honeybees and their associated viral pathogens provide best examples [59]. Multiple RNA viruses have been shown to be transmitted from managed honeybees to other pollinating insects as well as arthropods living in close contact with honeybees or sharing the same environmental resources [59,60]. To date, only two case studies involving potential ant virus spillover have been reported: (1) Nylanderia fulva virus 1, an RNA virus infecting the tawny crazy ant (*Nylanderia fulva*), was found infecting the red imported fire ant [61], and (2) Solenopsis invicta virus 4 (SINV-4) infecting the red imported fire ant was found in multiple co-existing ant species (although no evidence of active replication was found) [62]. These findings indicate the possibility of viral infection in a non-host ant. Thus, co-introduction of AgrV-3 with the ant host in the invasive areas such as Taiwan and Okinawa, combined with its high mutation nature, suggests that the risk of these viruses posing threats to local ant/arthropod community via virus spillover should not be neglected. Further investigation is warranted to test whether cross-species transmission of AgrV-3 or other RNA viruses associated with other global invasive ants [11,18,63] has occurred.

Lastly, our study also reveals potential limitations in short-read sequencing technology. The eight unmapped viruses were only partially characterized due to difficulties in assembling and recovering full-length transcripts associated with short-read sequencing [64]. This effect is predicted to be especially profound when viral loads are low, which appears to be the case for the unmapped viruses in the current study. Another caveat is that the assembly from a short-read library may generate artifact chimeric contigs because of the presence of highly similar variants (e.g., quasispecies). The hybrid sequencing which incorporates long-read high throughput sequencing (e.g., PacBio and Oxford Nanopore) likely resolve the issue as long-read sequences could provide a long reference backbone, whereas short-read sequences compensate the major drawback of high error rates associated with the long-reads platform [65]. Beside the read length, various bioinformatic tools for de novo assembly such as MEGAHIT [66] and metaSPAdes [67] have been known to mitigate the above-mentioned difficulties in assembly. Trinity assembler was selected in the current study for its minimum requirement of random access memory (RAM) and time-efficient nature while retaining similar de novo assembly accuracy. As the purpose was to verify the existence of chimeric contigs in our dataset, only PCRs and Sanger sequencing were employed. Long-read sequencing is currently underway to generate the complete genome sequences for these viruses with partial genome. We also note that our transcriptomic method may not capture DNA viruses, if any, in this ant, and a different sequencing library for high throughput sequencing should be established in the future to comprehensively understand the virome of this ant species.

## Figures and Tables

**Table 1:** Single-stranded RNA viruses and virus-like fragments discovered in *A*. *gracilipes*.

Family/Realm	Genus	Species	Average Read Depth *	Contig Number	NCBI Accessions
*Polycipiviridae*	*Sopolycivirus*	*Anoplolepis gracilipes* virus 3	95.73	18	MW078933
*Dicistroviridae*	*Triatovirus*	*Anoplolepis gracilipes* virus 1	50,371.26	2	MT108239 ^+^
*Dicistroviridae*	*Triatovirus*	*Anoplolepis gracilipes* virus 2	42.63	3	MT108240 ^+^
*Dicistroviridae*	unclassified	*Dicistroviridae* sp. 1	170.87	2	MZ394715 MZ394716
*Dicistroviridae*	unclassified	*Dicistroviridae* sp. 2	5.77	1	MZ394717
*Dicistroviridae*	unclassified	*Dicistroviridae* sp. 3	19.98	2	MZ394718 MZ394719
*Dicistroviridae*	unclassified	*Dicistroviridae* sp. 4	28.53	1	MZ394720
*Iflaviridae*	unclassified	*Iflaviridae* sp. 1	3.52	2	MZ394721 MZ394722
*Iflaviridae*	unclassified	*Iflaviridae* sp. 2	23	1	MZ394723
*Riboviria*	unclassified	*Riboviria* sp. 1	3.92	1	MZ394724
*Riboviria*	unclassified	*Riboviria* sp. 2	7.3	1	MZ394725

* Average read depth is the average support reads per nucleotide. ^+^ Complete virus genomes described in [18].

## Data Availability

All virus and virus-like sequences in present study are available in GenBank (Accession number: MW078933, MZ394715-MZ394725). Raw reads can be made available upon request to the corresponding author.

## Supplementary Materials

The following supporting information can be downloaded at: https://www.mdpi.com/article/10.3390/v14102161/s1. Figure S1: The fast minimum evolution phylogenetic tree based on the open read frame (ORF) detected in virus-like contigs. Table S1: Primers for virus verification in this study. Table S2: Pairwise sequence identity of amino acid between *A. gracilipes*-associated viruses. Table S3: The presence of AgrV-3 and other associated virus-like sequences in field-collected *A. gracilipes* colonies. Supplementary Data S1: *Anoplolepis gracilipes* virus 3 associated 18 contigs assembled by Trinity.
