# Supplementary material for: First Polycipivirus and Unmapped RNA Virus Diversity in the Yellow Crazy Ant, Anoplolepis gracilipes
Source: Viruses. 2022 Sep 30;14(10):2161. doi: 10.3390/v14102161 (PMC9612232; doi:10.3390/v14102161)
Supplement: Supplementary file 1 [file viruses-14-02161-s001.zip › viruses-1923334-supplementary.pdf]

viruses

MDPI

Supplementary Material

First Polycipivirus and Unmapped RNA Virus Diversity in the Yellow Crazy Ant, *Anoplolepis gracilipes*

Chih-Chi Lee, Hung-Wei Hsu, Chun-Yi Lin, Nicolas Gustafson, Kenji Matsuura, Chow-Yang Lee, Chin-Cheng Scotty Yang.

Table S1. Primers for virus verification in this study

Virus	Primer name	Nucleotide sequence* (5'- 3')	Target region	Fragment size	NCBI Accessions
Anoplolepis gracilipes virus 3	AgrV3_RdRp.for<br>AgrV3_RdRp.rev	CAAAGTGAATGACCCCGAGT<br>ATGCGAGAAATCGTGTTTCC	RdRp	756 bp	MW078933
Dicistroviridae sp. 1	DicSP1_RdRp_F.for<br>DicSP1_RdRp_F.rev	TAAGATTGCGGAAAGTGAGAAAAGG<br>TCCACTCGTGACATACTTCCTTTAG	RdRp	579 bp	MZ394715
Dicistroviridae sp. 1	DicSP1_RdRp_T.for<br>DicSP1_RdRp_T.rev	GCTCAAGATCACGTGTTTATTGA<br>AGCGAAATTCTGACATTGCTAGA	RdRp	724 bp	MZ394716
Dicistroviridae sp. 2	DicSP2_RdRp.for<br>DicSP2_RdRp.rev	AGGAATAGAAAGAATTATTGAGTGGGT<br>TCCATATCTGTCCAGCGAAATTCAT	RdRp	201 bp	MZ394717
Dicistroviridae sp. 3	DicSP3_RdRp.for<br>DicSP3_RdRp.rev	GATGCTGTTGGTGGTATTGGTATTC<br>ATTGTCGAGTTTGTTTCTGATCGTC	RdRp	439 bp	MZ394718
Dicistroviridae sp. 3	DicSP3_CP.for<br>DicSP3_CP.rev	TATGTTTCTGATTCACTTTTCCGCC<br>AATGACATCTCCTACCTTATTCGCA	Capsid	654 bp	MZ394719
Dicistroviridae sp. 4	DicSP4_RdRp.for<br>DicSP4_RdRp.rev	AAATCATCTATTACTTATCCCTTGGC<br>ACTTCTTCACTAGCCTCAAATATGGA	RdRp	608 bp	MZ394720
Iflaviridae sp. 1	IflSP1_RdRp_F.for<br>IflSP1_RdRp_F.rev	TACTTATGTACGGTTGTGTTCGGAA<br>GCTTCCTGTAATGTCAATTCACGAT	RdRp	310 bp	MZ394721
Iflaviridae sp. 1	IflSP1_RdRp_T.for<br>IflSP1_RdRp_T.rev	CGCTTCAGAGATAAGAATGTTATATTTCCT<br>TAATCTCCCGTTACTATATTATCACCAACT	RdRp	290 bp	MZ394722
Iflaviridae sp. 2	IflSP2_RdRp_F.for<br>IflSP2_RdRp_F.rev	TTCAAATTTCGGACCTTCTCTCTCT<br>ATGTCTGTTTGTTTATGGCACCAAT	RdRp	597 bp	MZ394723
Riboviria sp. 1	RibSP1_RdRp.for<br>RibSP1_RdRp.rev	TGAAGCCGTTGAAATAGAGGAAGTA<br>CCCGATATACCGTTAAGAACCTCAT	RdRp	450 bp	MZ394724
Riboviria sp. 2	RibSP2_CP.for<br>RibSP1_CP.rev	TGTTAGGTGTTGGTGGGATAGAATT<br>GAACAGGTCGAAGTATGAGAGGTAA	Capsid	687 bp	MZ394725
Agr_EF1a	Agr_EF1a.for<br>Agr_EF1a.rev	CCTGGGTGTTGGACAAACTT<br>GCTCCTTCACCGAGATGTTC	Elongation factor 1 alpha	789 bp	DQ226017.1

* The annealing temperature for all primer sets is 60°C

Table S2. Pairwise sequence identity of amino acid between *A. gracilipes*-associated viruses

Viruses	*Dicistroviridae* sp. 1 5'-end contig	*Dicistroviridae* sp. 1 3'-end contig	*Dicistroviridae* sp. 2	*Dicistroviridae* sp. 3 5'-end contig	*Dicistroviridae* sp. 3 3 '-end contig	*Dicistroviridae* sp. 4	*Iflaviridae* sp. 1 5'-end contig	*Iflaviridae* sp. 1 3'-end contig	*Iflaviridae* sp. 2	*Riboviria* sp. 1	*Riboviria* sp. 2
Dicistroviridae sp. 1 5'-end contig	100										
Dicistroviridae sp. 1 3'-end contig	Na	100									
Dicistroviridae sp. 2	Na	Na	100								
Dicistroviridae sp. 3 5'-end contig	Na	24.561	22.222	100							
Dicistroviridae sp. 3 3'-end contig	Na	33.333	Na	Na	100						
Dicistroviridae sp. 4	41.667	45.455	41.667	Na	Na	100					
Iflaviridae sp. 1 5'-end contig	Na	27.273	Na	40	44.118	Na	100				
Iflaviridae sp. 1 3'-end contig	33.333	28.358	Na	Na	Na	Na	Na	100			
Iflaviridae sp. 2	Na	26.102	Na	32	30.357	Na	79.487	81.944	100		
Riboviria sp. 1	Na	23.529	Na	Na	Na	Na	Na	Na	46.667	100	
Riboviria sp. 2	Na	23.611	25.806	23.529	35	Na	33.333	24.194	42.105	Na	100

"Na" indicated no overlap between contigs

Table S3. The presence of AgrV-3 and other associated virus-like sequences in field-collected *A. gracilipes* colonies

Viruses	Malaysia	Indonesia	Taiwan	Okinawa	Hawaii
Anoplolepis gracilipes virus 3	+	+	+	+	-
Dicistroviridae sp. 1	+	-	-	-	-
Dicistroviridae sp. 2	+	+	-	-	-
Dicistroviridae sp. 3	+	-	-	-	-
Dicistroviridae sp. 4	+	+	-	-	-
Iflaviridae sp. 1	+	-	-	-	-
Iflaviridae sp. 2	+	+	-	-	-
Riboviria sp. 1	+	+	-	-	-
Riboviria sp. 2	+	-	-	-	-

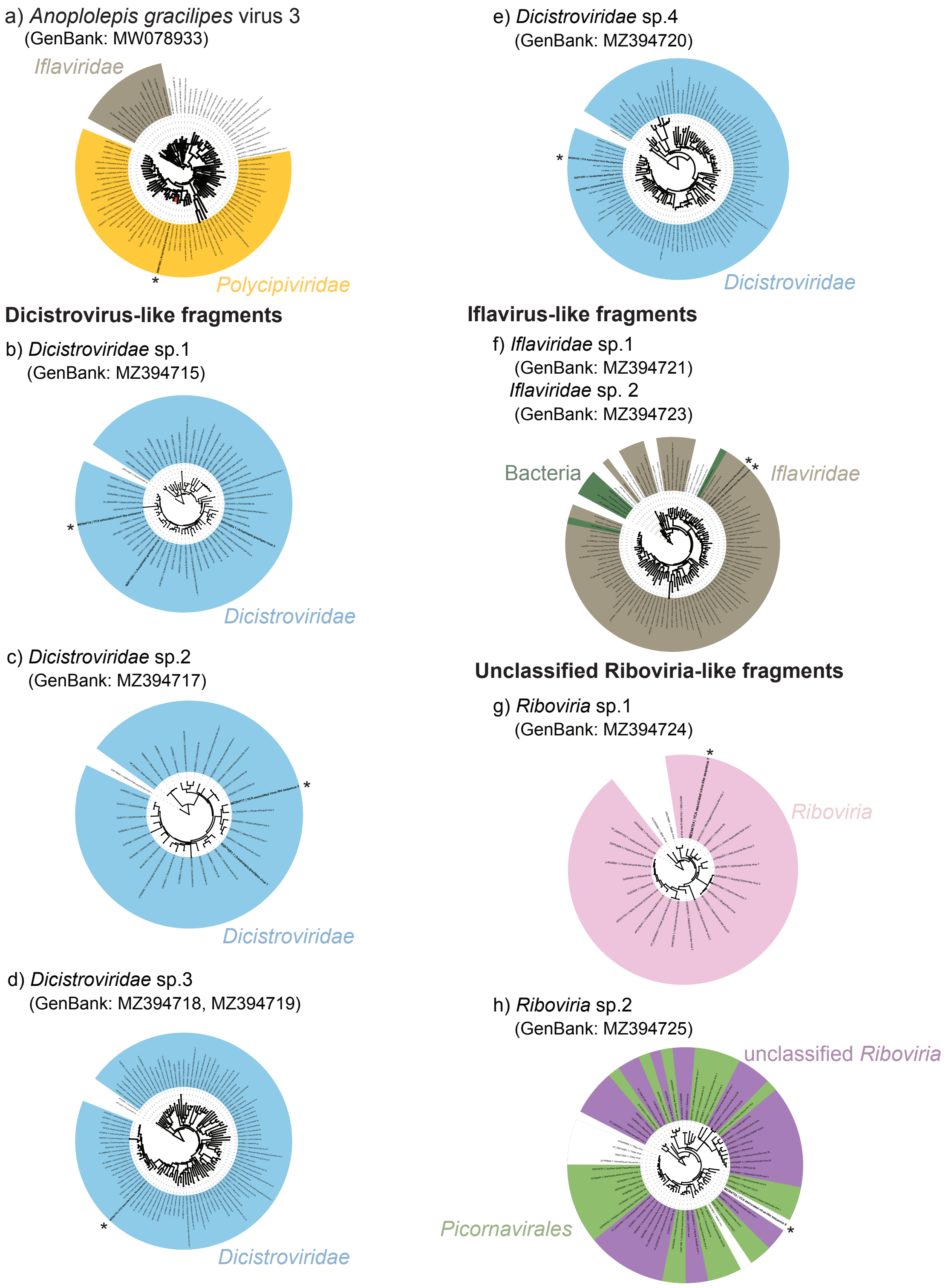

Figure S1. The fast minimum evolution phylogenetic tree based on the open read frame (ORF) detected in virus-like contigs. The star (*) denotes the respective virus-like contig found in *A. gracilipes* transcriptome.

Supplementary data 1: Sequences of 18 Anoplolepis gracilipes virus 3-associated contigs assembled by Trinity

>TRINITY_DN93_c0_g1_i4 (Representative, MW078933)

CATAGGGTCACCCCCTATGTATGCTTCACATTCTTCACGAAGATGTGTTCTTGGAGCTCTC-
TATCGCGTGTCTACGGACCCTTACATCTCCAATTATTTTAAAGAATGGAAATTCTAAAATTCAACTTTTGTATGCATGTATG
AATGAAAATTAATTTAATGGGACGAAGAAATTATGTTGATATGCCCATTATTTTAA-
TAACATCTTTAAATTATCATGAATATAATACAACCAACTGAAGAAAGCATCGACAAGAGTCGAGCAAAAGAAATTGGCC
ACGGTACTATACCCTTGCCAATGGAATCTAATATGCTTAATATACCATCAGCCCCTG-
GATCAGCTTTTGATGACAGGTCATCGGTCATTGCTACTAAATGGGCTTTTGAGCAGTTAGTCTCGCAGAAACAACTTATGA
CGACCATTGCAATTCCCACAAATGGAACCGGTACGACTCCCTTATTTC-
TATTTCAAAATTCTTGGAATAATATTCAAAAATTGCATTTTAGAAATTTATCAGATTTATTCTTTTTAAAGTCGTGGAAATGG
CATTTAACTTTTGAATTTCGATCAAATTTTCAGGAAGTTGGAATGATGAC-
TATTGCTTATGCTAACGTTCCACTTGATGCTATTCCTTATTTAACTTCAGCTCCTATTTCTTATGTTGATACACAGATCACTAA
AAATGATACTCCTGGACAAAATGGCCTTGTTACTGCAGCGAA-
TATAACAAATCATTCTTTATTTAGTTTAACATCCATGAATCAGTTGCCTCATGTTCATGTAATGTTGGGAGAAAACCAAGAT
GTAGAGTGTACATTTGATTGGCTTTCACCATTTAAATCATCTTTTACACGAATTAAAC-
CTTTTAATCCTAATTATGTATTTGGCGATAAATATCAAGATCCTAACGACCCGCTTTATGATATGGGCTTTGTTTATTTAGTT
AATACAATTCCGTTGACAACTGCTTCCGGTGTTACCCCACACGCTACAGTAAGAATTT-
GGTCACATCTTACCGATGTTGAATATTCTGGTTATGTTCCTCAAGATAACATTATATGAATTTTGTAAAATCATCAAATTCA
ACTTCTTTATCAGGCCCTAATGTTACCTGGACTACAAATCCAATTTAC-
CAATCTCCCGAAATGGCAGGAATATCGTGGTATGATCAGGTCAATCACTGGGATCCTCACTCAGAAGGTATATTTTCCATT
AGCCAGAATTCTACTAATGATCCTATTCCATATTGGACCAACCGTGAATCGGACTTATCAG-
GAGTTCGTTCACAGTGGTCAAGAATTGAAAAACAATCTGCGCAAAATGCACTTAGCCAAACGACGCCTGCGATACCGTCG
ACTAGTTCCGCGCCCCAATCTAGAGTCCCTATTGCCCCTCTGGCAGCAGCAGAAAC-
CAAAGGCCCTACAAAATTAGCTCAATCAAAGGACGTTGCAAAAGTTGCTAGCGCTGTTACAGGTGTTGGCAATGCTGCTTT
ATCAGTAGCCTCCTTAACTGGACCTGCTGGTATTGCTGCTG-
CAATTAATGCTGCCGCTGGTGCTGCTACTGCTAGTGCTATTGATGCTGGAAATAAATCAACTATAGCATCTGATTATGTAGC
AAATACTAAAGTTCAAGGATCACAATCAACTCATCAAGCACAACTTCTAAAGGAAATTGA-
TACCGCTCATGCTAACGTTACTGCTTCTGGCGCTCAAATTGGTGGACTTTTTGGTCCTGTTGGCGCGTGGTTGGGAGGTTCA
CTTGCTAATGCTATTCAAGACTCGGCACCCAAAGATACATATAATGATTTGAAAACAGGC-
TATTCATTTGAAGGTCGATTTAATCCACAAGATACTGGCTCTGTAAATTCTGGTACAACTGCTAATTTATCAGGTCAAACAA
ATATTCAATCTAATATATAATGTCATTAAATGAAGAAATTAATGCAGATACCCATAATCCAG-
TTCGAAATTATGGTTTGCCTCAGAACCCTGTACCTCAGACAAACACTGGGCTACTTGAACATACTGGAATACCATTTGAAC
CATTAGAGAACATTTCATCTTTACCACCACAATTCTCTTGGATGGTTGAACAG-
TATAAAATTTTAAGCACACAACAAGTTAGTACTACTAATGCGGTAGGATCCATTATAGTTTCGACCGATGTTTTGCATCCTA
ATAACGGTAATACAGTCTTTCGTACTTATCCAAATTGGGAAAGAATTCCTTTT-
GCTTCATCTATTTGGTGGAAAGGAATTGTTTCTTATCGTTTGTCTATTATTAAACCACCGCGTGTTACTGGAAAGCTTTTAGT
TCGTTACAGACAAGATAACTTCTTAGTTTTTAATGGTAGGCCAGCCAATAGTAGCAA-
TATTAAAGACAGCACTTATCGATCAATATTAAAGGAATGGGATTTATCTGATTCCAATCATTTTACTTTTGATATTTCAGCTT
CATTACCTATTAGGGCCCGTCCTACTA-
AATTTCCTGGCCTTAAAAGTATAACTCCTTCAACTGCACAAGATAGAGTTAGTTATGCCACTACGTTAACACCTTGGATCG
ATTTTTCAATGGGAGCAATTTCATTGGAGGTCGCTGAAACTATAGCGCCTGGCAG-
TATATTTCCGGATAGTTATACTATAATTATTGAACGAGCTTTTAAAAATACTGAATTTATGATTCCAACTGATTCAAAATCT

ACATATTCATTATGCGTTAAAGAATCACCCTTTTATACAGCACCATGAGTAACGAAGAACCG-
CAAGTAACTCCAGAACAAAATGTTCAATTGCCCAGCGAGTCTCATAAACTCCCTGAGCAATCTAAAATTCCACAATGCAA
TATTCCTAGGCCTTTTGATTTTGATCATCTTATACATCAATGGCAGCCCATGGGTATTAGAG-
TCACTTTAAATTTGCCTTTTACTGGAAATGATCAGGATTATATTTTTGCTATTCGAAATGGACCATTTATCCCTACGTTATTTT
ATCAATACAAAGATTCTTCTTGTGAAACAACCCTTGATCCTACCTCGAAACAACCTAG-
TATTCAGAATAGAAAGCTAACAACAATGGATACGTATCAGTGCTATGCTTTTAATAACATGCGTAACGTAACACATGCTG
GTAAAACTTATACACAAATTGACCCAGATCAACAAGCTGTTTATATTACTCAA-
TATGATGCACCACCAATTTTAGCTTCATTAGCCACTATGTTTAGGAAATGGCGAGGAACTATGCATTATAGAATTCGTACA
GTAGCAGGATTCACAACACAAGGATATATATTTTCATCTGTAGTTCG-
TAATTCCCCATCCGTTGTCGGTATTTATCCTACTGAGGACACTACACCTGGAATTCAAAGAGAAGATCGGTCTTACCGAGA
ATCTATGATAAATTCGTATGTTATGGGTGATACGGCAATGTTTCGTCATTTTGAGGTT-
GAAGTTCCCTTTGAATACCCTGTTCCATTTTATGATCAATTTAATTGGATTGGTAATCGTTCTCGTCCTGCAAAAAATTTTAT
GACTTGTGATGTTAATGGAACAACTCGAGTCCAACGTCTTCGAAATATTCATAATGAAC-
CTCATGGAGATAATTATATTTTGTTTGGCCTTCGCGGTAAATTGGAGTCTTCTGTACAAAATTCACAAATAACATTTGAACT
TGAATACCGAGCAGGGGACGATTTCCAATTTGCTGATCCGTTTTTACCTTATAATGCTCAC-
TTTTTAACTCCTTATTCAACCTATTCAGATAATAAGACAAGTATTATTCAGGTTCCTTCTAAAGATTATTTTACTGACGGACT
TCATGATTATTCATCAACACCACTAACACAACCTACTATAGCGCCTGTTGTAG-
TTCGTGATCAAAATCCTGCAGACACAGTTGCTACAAGACCTAAGCAAAATACTATGCAAGCTATTCCTCGATATGATCCTG
TAGAAACGAATGTTTCAAGGCCACGAAATCCTCGCCCTCGTACAATTAGAGAT-
TTAACTTCATATGATACGGTTGATTATAAGCCCGTTTTGCGAGATGAAGATCTCGTAAGCGAGCCTGGTGACGACTTGGAC
CTCTTAGACTTGAAAAGGAATCTTCGAAAGTCTTTGAGAGTTGGA-
GATGATGTATCACATTTATTGCAAAGTTAGACATGTATTATGTGCAATAAGATTGGATACTGATCTATAGCCTGGAGACTT
ATGTTTACTATTCAACAGACAGTCTGGCACAATGAATAGCTCCCGTTTGGTT-
GGTCTCTGTTTTACGGATTAAAATCGGGACCCGTTACTGTAGTTCGTAATACTACACTTCAGGTTAGGCTCTTATGCGCTAA
GAGTACAAGCACCAGTCAATTGGTTACCTGTCGTAGGACCTAACGGTAAGAGGAACGGCAA-
TATGGCGATCCCATGCTTAGTACTCAACTCAAAATATGAATATGTTCTCAACTCAAACTCAATTTACTACAAAACAAAACA
ATGAAGGAAGAAAGAAATTTGGAATTGGAAACAAGATTTCCAACGAGCTTAGACGAG-
TCATCAGACAATCGACCAACTGGGTATATCATACACCCGCTGATCTCCTCAAGCTTTTTGAATACGATTCAAACCGTCGAT
ATATGCCTGTACGTGGTAATACTATTGTGCGTTTCAATTACAC-
TCTTAGCGATGTCTTATCGCTTATGCGTACGTTCCAGATACAGTTTAAGGAGTCACATCACGGCAGAAAACTCGCTTTATCG
CCCATATTCTCCAAACCAGACTCTACCAGCGTAGTAGATCAAGAATTAGATCAA-
TATGACTCATTCCCATATTTCAGTGCTATTAATTCACATGATTCTGCACTGTGTAACACCGTATCCATCATGGAGGAATTTTC
TTCTCGGCAAACTTATAATATTAATGGAGCATTTCGATATGCTATTGCTGACAC-
CTACATGACATGTTTGTATGAATTTTTAAATTTGTTAACAACTACATCAAAGAGAGATATTGAAGAAAAATTGTTTACACA
TTTTAATCATGTACAATTCAAAAGATTAGATGCTTATGGTGTTTACATGTTTTT-
GCTTCATCTTCGCGTTCGTGGAGTTGCAAAGTTAGATTGGTATTATAAATATCAAGCGTGGCGCAAGGGATGGGCATCTTA
TAATATAATTGAAAAACAACAAATTTTAAATTCATTTTATAAGGGCGAAAATTCAC-
GTATTTTTAACACCATGCAGGAGATGATGATTGATGAGCGCAAATGGTTTTATTCCATTTTTGCTCATGAAGCAAAATTAAT
TCGTTATTCAACAAAAATTACAGGTCAATTATTTAATCCTAGACTTATTTATTATTTAACTT-
GGAAAAATGCTATTTTAGATCAAATGACACCTACGGAGAGATATAGAGATTCATTGCAAATGCCTCTGTCACATTTACATG
GTGAGCCAACCATGTTTCGCATTAAAGATAACATTAATGAGGCCGTGGATGAGAA-
TATTCCTAAAGTTCAATCAGCGATACAGGATTCCTTGAAGGAATTGTTAAAATCCCCTGAGATAAAAGAGTCTGTTACTAA

TTTAGTTACTGATTCAATGATCCCTACTCTTGAAAATTTTGAAAAGTCTTCTGA-
GAAAGTTAGTAATTCTGTTTTAGATAATTTGAAAACTTCAATGGCCCCTCTAATTGAACAAACTTTTTCTTTGTTTTCGACAG
TTAATGGACTAATAGATTTTATGAAATCTATGCTTAAGCAGGCTCTT-
GATGCTTTCCCAAAGGAGCTTTTCGGAACCAAGATGAATCTCGACATTTCTCCAGAAACATTGCTGAATCTTCTTAAGTATT
ATATTATTTATGTTAATGTAGAGTCTAAACCTTTAAAAATAGCTTTGATATATCTTATGC-
TACGAGAAGTGGGCCTTCTTGAATATTTAATCCAATGGGGCGGTAATATATTTAATCTTGCGTTTGGATCACGTAGTGAAGT
TCCATTGGATGGATCTGAAATAAAAGGCGAACCAACATCTGGCATT-
GATTGGCTTTCAAATTTGGCAGACATGATTTTTGGTCATAAAAATGAAATTGCTTTATGTTCTATGTTTACAGCTTTATTAGT
TATGATTTTTAAACATACTTGCGGTCTTCGTAATGCAGGAACCATGCGTTTTAATGAA-
TATACAACAATTGCTGGCATGGTTATTGGAATGTGCAAAGGTTTCCATTGGGTTGGAAGCGGTATGTTTGGCATTGACCGT
ATTTATAAATATTTTGTTATTATTTCAAAATCGGTTACATCTTACATCCAA-
GAACATATTTTAGGTATTACTGAACAAGCCATGACTAATGAGAAGGCAGTGGCTAGGTGGCTTGTTAAGCTCAAATTTTTC
TCCACTGATACAGGACGCAATGCAATTCGTGTTTCAGAAGATCTTTTAAAAGTTGCAGAGCG-
TATTATGTCAGAAGGTCTCGCTTTTCTAACAGCTTGTTCAAAAGATCCTAAATTTATTTCTCGCGAATCTTTAATGGTCATTC
ATCGTAATTGGAATGATGTTAAGACTTTATCTAATTATACTTATCG-
TATTCGATCTACTTCTAATTTTAAACCTGCAATGTTTCATGTACAGTTTGTAGGGGAACCTGGTATTGGAAAATCAACTTTA
ACGGAGAAGTTCATTGCGTCATTATCTAAGAAAATTTATCGCGAGGATAAAAA-
TATAACCCATTGGACTTATAATCCTAATGTAGATCACTTTGATGGTTATAATGGTCAAACATATATGATTATCGATGATCTT
TTTAGGTACAATGAGCCCAAACATTTATCTTTAGTTATAGGACTTATTACTAATACACCAG-
TCCCCCTTCCTATGGCTCACTTAGAGGATAAAGGAGTTCATTTGGATTCGGATATTTTAATCTCTTCTACTAATGTTCCCTAT
CCAATCGGAAAAGACATATTTTGCATGGAGGCTGTTCACAGAC-
GTCGTCATGTATTGTGTGAAGTCAAAATGGACCCTCGTGTTAAAGCAGATGGTAAATTTTCAAAACAACTTTTTGAGAAAT
TTTATCCGAATCAAAATTCGTTTGATTTTCCTCATCTTAAATTTGCTTTAATGAAAC-
CTGTTATCAATCCTGGTGAAGAGCAATATCAGAGTACAACTTCTGATGAGATGGAGTGGAAGCATAATTTAATTAAGAAA
CTTCGCACAGCTAATGAAACTCTAAAATTTAATGAA-
GACTTTTTCTTCGGTCCCGATGCTCGCCCTTTGAAAGGAATGACAGTTCCTTGTACTAACTGGACATTTGATACTTTTGTTGA
AAATGTAGCGGTTAGTTATCAACATCTTAGGTCAGGAGAGCAAAAACTAACAG-
TTAAGGAAAAATATGAACATGTTATGGAATGCTTCGCTGAAATTGACAATATATTTGTTCAATCAGATGATATCTCAGACG
GTGTGGCTGCATCAACCACATTTAAACTTATTTCTGATAAATTTTTAGATGCTTCTCTTCAA-
TATGGCATGGATGATCCTCTTGGAAAGCGTGTTTATGCTCAGACTTTAGATCAAGTACTTCCAGACATGTGTGATCTCGACA
TCGATGGTATAACTCAGGAGTTTTTAGATTCTACAGAAGGTATGCCAACTGCCGGTAC-
CGTCGATGATGATGATTGGGAATCTAGTTATGGTGACCCAAATGAATTTTTGGGTCTTACTTTAACAGATGAAGAGATTAA
ACTTGAAAAACTGACTGAGCACATTCGATCTCATGATTTGACAGGACCCGAACGCGATTTAG-
TCTTATTGCTGATGCATAAGTGTGCCATGAAAGAGCATTTCACAATTTCAGATTATCGCTACTTTTCATATTTAGATGGTAA
GGCACCAAATCCTTTTCCTAAAGAAGCCTATCAAGATATTGTTCCTGG-
TAATCCTGGCCCTGAAACATCTAGACGTCAACGTATTATGCAAAAATTAAATAAACGTATTATTGATCCCTTGATTAAAGA
GCAGATGGTTGTCAAATTAGTTCATAATTCAGAAGGCAATCTTTATCCAGCTATACCAATTT-
GGTCCTATTATACTAATTGGGGTTGTTCCGAATCAGGAACTTTCCCTGGAGAAAATTTTTATAATTATTTGCAATCAGATCA
AGTAATGGAATCATCTTATCAAAATTATAAAACGCGATTTATGTGGGATAAAC-
CGAAAATGATGTCCTTTTTCAAACAAGTAAATTCAGAGCACAAATTTTATTACCCTAATCGTAAACCTTATTCTGATGAAC
ATATTGAGCAAGGAAATTCACGAATATCCATTGAGTTTCTTCGTCGCCTTAGCTATGTT-
GATGGCGATTGGCATATGGATGTATCAGATCTTGATTTTCATATTACAAATTCTATAGCGATTAAGAAAAATTTGAATAAC

ATTCAGAAAACTTATCATATACCTTTAGATATAGCATTTTTCTGCTC-
TATAAATCAATCATTTACTTATACAGCAAATTTATTTCATTGCTCTCAGCTGAGGAACAGCGTGATATGGTTTCAGCTGCC
AAATGGCAATATGAACATATGTATTCTTTATCACGAGAACATCTTAGA-
GAGCGAATTTTATCGATAGTCCATACAGTGAAAAGAGTTACCCTAGCTCATGTTTACCGCGCTGTAGGTTGGGTCTGGAAT
AAGCTTTCTACAATTGTCTCTTCATTTTTAAAAATTGCAGCATTTGCGGGAAC-
GGTTTATTTACTTAAACAAGTGGGAAAACTGTTCTTCGGCTCATCTGAACCCACTTCCAAATTTATGCATCGCTCAAACATA
AAATCAAACTTGATGTTCAGAGGTAGACCACAAGCAGGAATTTTTGATCCATCCAATAC-
GCAACAAATTTTAGCCCAAAAATATTTAGATAAGCATATTAAGTTTTTCCACATTGTTAATGAGGATGGCCTTGATGTTAA
GGCTCATGGAATTCATACAAAACAATTTTTGATTTTAAATTCACATACTA-
CAATCCACATTAAAGGCCCAACTATATTGTCTTACACCCCTACAGTTAATTCTGATAAAGCATGGGAAATCGAGATATCCC
GTGATAATGTTTTTGAATCCCCTGGAAATGATCTTGCAATTATTTTTTCTCGTCACTTAC-
CTATGGCCTCTGATATAACTAATCAATTTATTACTAATGAGGATTTCGAACAATCTGAAAATATTGGCGAACTTTGGTCTCT
TACAAACTATGAAAATCAGCAATCTGTTGAGATTCGTGATCGTTGTGTCCCCCATAA-
GAAAGTCACCATGACTGCTAATGATGGAGCAAGAGGAGAAATGTCAATGGCTATCATGGTTGAGGGAGTGACAGTTGGA
GGAAAGAGTGGTTCAATGCTAATGTCAGCTAGTAAGCGTCCAGGACATCGTTCAA-
TAGTAGGTATTCAAGCCTGGAAAGTATCAGATTATTATAAGCAGACCATAGTTTATCAAGTAGTTACTCAAGAACTTCTTT
CCGAATTAATGCAACAGGTTGAAAAACAAGTAAAACGTCCTGTTATAACCCAA-
GAAGGCCCCATTTTATGTGAACCCACATCTTCAAAAGTGGAAGGTATTGTTGACTCTCACATCAACATTTGTGGTTCGGTTC
CGGCTGAACATGTTGTTGGTATGGTAGCTCGAACTGCCTTCAA-
GAAAACAATTATTGCCCATGAGATGGACAAGTCAGGAAAAACATCTCCTCGTGTTCCAGCTGCTTTGAATCCTCATGATCA
TCGGCTTTTAGTTCATGCTCATCCTATGCAACATTCAGTCAATAAGCATGGCACAG-
GAAAGGTTGGATCTTTTGATCTAGCAATTTTGAACCGAGCCACTGAAGATATGGCATATTGGCTGCGTGACAGATTAGACA
AGCAATCTTTTGATTGCAATTTGGATTTTGAAACCTGTGTTACGGGTATTCGAGAAC-
CAGGCAGTAATCCAGTCAATTGTAGTAAGTCAGCTGGCCTCCCATATGTATTAGATAAATATCATGGAAAACCAAAAGGC
AAGAAAGCCTATGTCGAAATTACTGAAGAAGGCCAATGCAAAGTGAATGACCCCGAG-
TTTATTAAATCATTTGAAGCCACTTTTTCAAAATTGCAACAAGGCATTATTCCAAAGCATTCATCATATGATTTTCCAAAAG
ACGAGCTTCGTCCATATTATAAAGCACTTGGCGATCCAGTTGCAG-
GAACTCTCCCTAAAACAAGATCTGTCACATGTATGAACATGGAAATGATATTTTCATGGCGTCGTGTGACTCTAGATTTGA
TGGCGTCTTTACATCGCGCTGCTCGTGGAGATTTTCCTTTTGGTCCTGGGAT-
TAATCCTGAGGGCCCTGATTGGACTCGCCTATTTCATTACTTGAATAAACACCCTAATTGTTTAGACTTTGATGTAAGTAAT
TGGGATGGCCATATGCCACCCGAATTATTATTATCCG-
TAGCTGACATGTTATGTATAATCCTTCGTGTTTCTTTTGATTCTCCTACCGCTAAAGTTATTTACTCATTATTAACCGAAGTTT
TGTTTGGCCATGTCCAATTCGAAGATATGGTTTATCAAAAATGTAGAGGGTTGATTTCGG-
GATTTCCTGGAACTGCCGAAGTCAACACTCTCGTTCACTTATTACTCATGTATTATTTTTATCTTTATATTGCCGATTTAACA
GAAAACCAACAATATGCAAATATTCATGATTTCTTTTATTATACATCACCTGTCTTCTAC-
GGGGATGATGTTATTATGTCAGTTGCTGAACATATTATACCATGGTTTAATGGAAACACGATTTCTCGCATGTACATTGAGC
ATGGATATCCTGTCACAACTGCTTCAAAAACCAAAGAGATGCCTAAAAGTAAAAA-
TATTTTCGAATGTACTTTTTTAAAATCCGGTTTCAACTATATAAATCCATCTCGAGTGGATCGCTTGATGGATATTTCAGTTT
GCTATGATTTAATGTATTGGGTGCGAGCAAAAGAACACCCTTAC-
GATCAATTCCGTTCAAATCTCTTTGATTCTTTTCGTTTAATCCACGGTCATGGCCCCAATAAATATAATGAGGTAAGGGACG
AAGTTAACCAATGGTTGCGTGAGTGCAATCTTGAGCCCTTTGACTATCGATGGGAGGAT-
TTCGAAAAAGATAAAATTCAAAAATACTATTCAGAATAATTATTTTATTTTTAAGTTAGTATTATACCCGTAATGTAATTTA

ACGGGATATAAGGTAGGTCTGTTCCGATCGTCGCATTGTGAGACTAGCTGAAACCCTCTCG-
GAGGCTGGATGCTAGTTACTGATCCGCTTGCGGATAGTGGAACAGCTATTTTATTAAGTTAATTCTTAGACATGTAACAGT
GTATTTAGATCCCCCGTGGCTGGGCATAGTTTAAGTTTTGGCAATTT-
GTTTTAACATCTAAAGGCAGAGGCGTCTAAACTAAATTACCTCTAACCTATTTGTTGCTTAGAACTTGTTTCGTAAGTTCTA
AAAGCACGGATGCATTGGATTCTAGTGAAGCCACCATCGGGCATGAAGGTGC-
TACCCGTGTTTTTCTTTTCCATTTTATTAATTATTAATTATTATTATAATTATTATTATTATTATTATTATTATTTAGTTTTAAAA
AAAAAAAAAA
>TRINITY_DN93_c0_g1_i6
TTTTTTTTTTTTTAAAACTAAATAATAATAATAATAATAATAATAATTATAATAA-
TAATTAATAATTAATAAAATGGAAAAGAAAAACACGGGTAGCACCTTCATGCCCGATGGTGGCTTCACTAGAATCCAATG
CATCCGTGCTTTTAGAACTTACGAAACAAGTTCTAAGCAACAAATAGGTTA-
GAGGTAATTTAGTTTAGACGCCTCTGCCTTTAGATGTTAAAACAAATTGCCAAAACTTAAACTATGCCCAGCCACGGGGGA
TCTAAATACACTGTTACATGTCTAAGAATTAACTTAATAAAATAGCTGTTCCACTATCCG-
CAAGCGGATCAGTAACTAGCATCCAGCCTCCGAGAGGGTTTCAGCTAGTCTCACAATGCGACGATCGGAACAGACCTACC
TTATATCCCGTTAAATTACATTACGGGTATAATACTAACTTAAAAATAAAA-
TAATTATTCTGAATAGTATTTTTGAATTTTATCTTTTTCGAAATCCTCCCATCGATAGTCAAAGGGCTCAAGATTGCACTCAC
GCAACCATTGGTTAACTTCGTCCCTTACCTCATTATATTTATTGGGGCCATGACCGTGGAT-
TAAACGAAAAGAATCAAAGAGATTTGAACGGAATTGATCGTAAGGGTGTTCTTTTGCTCGCACCCAATACATTAAATCAT
AGCAAACTGAAATATCCATCAAGCGATCCACTCGAGATGGATTTATATAGTTGAAACCGGAT-
TTTAAAAAAGTACATTCGAAAATATTTTTACTTTTAGGCATCTCTTTGGTTTTTGAAGCAGTTGTGACAGGATATCCATGCTC
AATGTACATGCGAGAAATCGTGTTTCCATTAAACCATGGTATAATATGTTCAG-
CAACTGACATAATAACATCATCCCCGTAGAAGACAGGTGATGTATAATAAAAGAAATCATGAATATTTGCATATTGTTGG
TTTTCTGTTAAATCGGCAATATAAAGATAAAAATAATACATGAGTAATAAGTGAACGAGAG-
TGTTGACTTCGGCAGTTCCAGGAAATCCCGAAATCAACCCTCTACATTTTTGATAAACCATATCTTCGAATTGGACATGGC
CAAACAAAACTTCGGTTAATAATGAGTAAATAACTTTAGCGGTAGGAGAATCAAAA-
GAAACACGAAGGATTATACATAACATGTCAGCTACGGATAATAATAATTCGGGTGGCATATGGCCATCCCAATTACTTAC
ATCAAAGTCTAAACAATTAGGGTGTTTATTCAAGTAATGAAATAGGCGAG-
TCCAATCAGGGCCCTCAGGATTAATCCCAGGACCAAAAGGAAAATCTCCACGAGCAGCGCGATGTAAAGACGCCATCAA
ATCTAGAGTCACACGACGCCATGAAAATATCATTTCCATGTTCATACATGTGACAGATCTT-
GTTTTAGGGAGAGTTCCTGCAACTGGATCGCCAAGTGCTTTATAATATGGACGAAGCTCGTCTTTTGGAAAATCATATGAT
GAATGCTTTGGAATAATGCCTTGTTGCAATTTTGAAAAAGTGGCTTCAAATGATTTAA-
TAAACTCGGGGTCATTCACTTTGCATTGGCCTTCTTCAGTAATTTCGACATAGGCTTTCTTGCCTTTTGGTTTTCCATGATATT
TATCTAATACATATGGGAGGCCAGCTGACTTACTACAATTGACTGGAT-
TACTGCCTGGTTCTCGAATACCCGTAACACAGGTTTCAAAATCCAAATTGCAATCAAAAGATTGCTTGTCTAATCTGTCAC
GCAGCCAATATGCCATATCTTCAGTGGCTCGGTTCAAAATTGCTAGATCAAAAGATCCAAC-
CTTTCCTGTGCCATGCTTATTGACTGAATGTTGCATAGGATGAGCATGAACTAAAAGCCGATGATCATGAGGATTCAAAGC
AGCTGGAACACGAGGAGATGTTTTTCCTGACTTGTCCATCTCATGGGCAATAATT-
GTTTTCTTGAAGGCAGTTCGAGCTACCATACCAACAACATGTTCAGCCGGAACCGAACCACAAATGTTGATGTGAGAGTC
AACAATACCTTCCACTTTTGAAGATGTGGGTTCACATAAAATGGGGCCTTCTT-
GGGTTATAACAGGACGTTTTACTTGTTTTTCAACCTGTTGCATTAATTCGGAAAGAAGTTCTTGAGTAACTACTTGATAAAC
TATGGTCTGCTTATAATAATCTGATACTTTCCAGGCTTGAATACCTACTATTGAAC-
GATGTCCTGGACGCTTACTAGCTGACATTAGCATTGAACCACTCTTTCCTCCAACTGTCACTCCCTCAACCATGATAGCCAT

TGACATTTCTCCTCTTGCTCCATCATTAGCAGTCATGGTGACTTTCTTATGGGGGACACAAC-
GATCACGAATCTCAACAGATTGCTGATTTTCATAGTTTGTAAGAGACCAAAGTTCGCCAATATTTTCAGATTGTTCGAAATC
CTCATTAGTAATAAATTGATTAGTTATATCAGAGGCCATAGGTAAGTGACGAGAAAAAA-
TAATTGCAAGATCATTTCCAGGGGATTCAAAAACATTATCACGGGATATCTCGATTTCCCATGCTTTATCAGAATTAACTGT
AGGGGTGTAAGACAATATAGTTGGGCCTTTAATGTGGATTGTAG-
TATGTGAATTTAAAATCAAAAATTGTTTTGTATGAATTCCATGAGCCTTAACATCAAGGCCATCCTCATTAACAATGTGGA
AAAACTTAATATGCTTATCTAAATATTTTTGGGCTAAAATTTGTTGCG-
TATTGGATGGATCAAAAATTCCTGCTTGTGGTCTACCTCTGAACATCAAGTTTGATTTTATGTTTGAGCGATGCATAAATTT
GGAAGTGGGTTCAGATGAGCCGAAGAACAGTTTTCCCACTTGTTTAAGTAAATAAAC-
CGTTCCCGCAAATGCTGCAATTTTTAAAAATGAAGAGACAATTGTAGAAAGCTTATTCCAGACCCAACCTACAGCGCGGT
AAACATGAGCTAGGGTAACTCTTTTCACTGTATGGACTATCGATAAAATTCGCTCTCTAA-
GATGTTCTCGTGATAAAGAATACATATGTTCATATTGCCATTTGGCAGCTGAAACCATATCACGCTGTTCCTCAGCTGAGA
GCAATGAAAATAAATTTGCTGTATAAGTAAATGATTGATTTATAGAGCAGAAAAATGCTA-
TATCTAAAGGTATATGATAAGTTTCTGAATGTTATTCAAATTTTTCTTAATCGCTATAGAATTTGTAATATGAAAATCAAG
ATCTGATACATCCATATGCCAATCGCCATCAACATAGCTAAGGCGACGAA-
GAAACTCAATGGATATTCGTGAATTTCCTTGCTCAATATGTTCATCAGAATAAGGTTTACGATTAGGGTAATAAAATTTGTG
CTCTGAATTTACTTGTTT-
GAAAAAGGACATCATTTTCGGTTTATCCCACATAAATCGCGTTTTATAATTTTGATAAGATGATTCCATTACTTGATCTGAT
TGCAAATAATTATAAAAATTTTCTCCAGGGAAAGTTCCTGATTCGGAACAACCCCAATTAG-
TATAATAGGACCAAATTGGTATAGCTGGATAAAGATTGCCTTCTGAATTATGAACTAATTTGACAACCATCTGCTCTTTAAT
CAAGGGATCAATAATACGTTTATTTAATTTTTGCATAATACGTTGACGTCTA-
GATGTTTCAGGGCCAGGATTACCAGGAACAATATCTTGATAGGCTTCTTTAGGAAAAGGATTTGGTGCCTTACCATCTAAA
TATGAAAAGTAGCGATAATCTGAAATTGTGAAATGCTCTTTCATGGCACACTTATGCATCAG-
CAATAAGACTAAATCGCGTTCGGGTCCTGTCAAATCATGAGATCGAATGTGCTCAGTCAGTTTTTCAAGTTTAATCTCTTCA
TCTGTTAAAGTAAGACCCAAAAATTCATTTGGGTCACCATAACTAGAT-
TCCCAATCATCATCATCGACGGTACCGGCAGTTGGCATACCTTCTGTAGAATCTAAAAACTCCTGAGTTATACCATCGATG
TCGAGATCACACATGTCTGGAAGTACTTGATCTAAAGTCTGAGCATAAACACGCTTTCCAA-
GAGGATCATCCATGCCATATTGAAGAGAAGCATCTAAAAATTTATCAGAAATAAGTTTAAATGTGGTTGATGCAGCCACA
CCGTCTGAGATATCATCTGATTGAACAAATATATTGTCAATTTCAGCGAAGCATTCCA-
TAACATGTTCATATTTTTCCTTAACTGTTAGTTTTTGCTCTCCTGACCTAAGATGTTGATAACTAACCGCTACATTTTCAACA
AAAGTATCAAATGTCCAGTTAGTACAAGGAACTGTCATTCCTTTCAAAGGGCGAGCATCGG-
GACCGAAGAAAAAGTCTTCATTAAATTTTAGAGTTTCATTAGCTGTGCGAAGTTTCTTAATTAAATTATGCTTCCACTCCAT
CTCATCAGAAGTTGTACTCTGATATTGCTCTTCACCAGGATTGATAACAGGTTTCATTAAA-
GCAAATTTAAGATGAGGAAAATCAAACGAATTTTGATTCGGATAAAATTTCTCAAAAAGTTGTTTTGAAAATTTACCATCT
GCTTTAACACGAGGGTCCATTTTGACTTCACACAATACATGACGAC-
GTCTGTGAACAGCCTCCATGCAAAATATGTCTTTTCCGATTGGATAGGGAACATTAGTAGAAGAGATTAAAATATCCGAAT
CCAAATGAACTCCTTTATCCTCTAAGTGAGCCATAGGAAGGGGGACTGGTGTATTAGTAA-
TAAGTCCTATAACTAAAGATAAATGTTTAGGTTCGTTATATCTAAACAGATCATCAATGATCATAAAAGTTTGACCGTTAT
AGCCATCGAAATGATCTACATTAGGATTGTAAGTCCAATGGGTTATGTTTTTATCTTCGCGG-
TAAATTTTCTTAGACAAAGATGCAATAAATTTTTCAGTTAAAGTAGATTTTCCAATTCCAGGTTCACCAACAAATTGGACAT
GAAACATTGCAGGTTTAAAATTAGAAGTAGATCGAATACGATAAGTATAATTAGA-
TAAAGTCTTAACATCATTCCAATTACGATGAATGACCATTAAAGATTCGCGAGAAATAAATTTAGGATCTTTTGAACAAGC

TGTTAGAAAAGCGAGACCTTCTGACATAATACGCTCTGCAACTTTTAAAA-
GATCTTCTGAAACACGAATTGCATTGCGTCCTGTATCAGTGGAGAAAAATTTGAGCTTAACAAGCCACCTAGCCACTGCCT
TCTCATTAGTCATGGCTTGTTCAGTAATACCTAAAATATGTTCTTGGATGTAAGATGTAAC-
CGATTTTGAAATAATAACAAAATATTTATAAATACGGTCAATGCCAAACATACCGCTTCCAACCCAATGGAAACCTTTGC
ACATTCCAATAACCATGCCAGCAATTGTTGTATATTCATTAAAACGCATGGTTCCTG-
CATTACGAAGACCGCAAGTATGTTTAAAAATCATAACTAATAAAGCTGTAAACATAGAACATAAAGCAATTTCATTTTTAT
GACCAAAAATCATGTCTGCCAAATTTGAAAGCCAATCAATGCCAGATGTT-
GGTTCGCCTTTTATTTCAGATCCATCCAATGGAACTTCACTACGTGATCCAAACGCAAGATTAAATATATTACCGCCCCATT
GGATTAAATATTCAAGAAGGCCCACTTCTCGTAGCATAAGATATATCAAAGC-
TATTTTTAAAGGTTTAGACTCTACATTAACATAAATAATATAATACTTAAGAAGATTCAGCAATGTTTCTGGAGAAATGTC
GAGATTCATCTTGGTTCCGAAAAGCTCCTTTGGGAAAGCATCAAGAGCCTGCTTAAGCATA-
GATTTCATAAAATCTATTAGTCCATTAACTGTCGAAAACAAAGAAAAAGTTTGTTCAATTAGAGGGGCCATTGAAGTTTTC
AAATTATCTAAAACAGAATTACTAACTTTCTCAGAAGACTTTTCAAAATTTTCAAGAG-
TAGGGATCATTGAATCAGTAACTAAATTAGTAACAGACTCTTTTATCTCAGGGGATTTTAACAATTCCTTCAAGGAATCCT
GTATCGCTGATTGAACTTTAGGAATATTCTCATCCAC-
GGCCTCATTAATGTTATCTTTAATGCGAAACATGGTTGGCTCACCATGTAAATGTGACAGAGGCATTTGCAATGAATCTCT
ATATCTCTCCGTAGGTGTCATTTGATCTAAAATAGCATTTTTCCAAGTTAAATAATAAA-
TAAGTCTAGGATTAAATAATTGACCTGTAATTTTTGTTGAATAACGAATTAATTTTGCTTCATGAGCAAAAATGGAATAAA
ACCATTTGCGCTCATCAATCATCATCTCCTGCATGGTGTTAAAAATAC-
GTGAATTTTCGCCCTTATAAAATGAATTTAAAATTTGTTGTTTTTCAATTATATTATAAGATGCCCATCCCTTGCGCCACGCT
TGATATTTATAATACCAATCTAACTTTGCAACTCCACGAACGCGAAGATGAA-
GCAAAAACATGTAAACACCATAAGCATCTAATCTTTTGAATTGTACATGATTAAAATGTGTAAACAATTTTTCTTCAATAT
CTCTCTTTGATGTAGTTGTTAACAAATTTAAAAATTCATA-
CAAACATGTCATGTAGGTGTCAGCAATAGCATATCGAAATGCTCCATTAATATTATAAGTTTGCCGAGAAGAAAATTCCTC
CATGATGGATACGGTGTTACACAGTGCAGAATCATGTGAATTAATAGCACTGAAATATGG-
GAATGAGTCATATTGATCTAATTCTTGATCTACTACGCTGGTAGAGTCTGGTTTGGAGAATATGGGCGATAAAGCGAGTTT
TCTGCCGTGATGTGACTCCTTAAACTGTATCTGGAACGTACGCATAAGCGATAAGA-
CATCGCTAAGAGTGTAATTGAAACGCACAATAGTATTACCACGTACAGGCATATATCGACGGTTTGAATCGTATTCAAAA
AGCTTGAGGAGATCAGCGGGTGTATGATATACCCAGTT-
GGTCGATTGTCTGATGACTCGTCTAAGCTCGTTGGAAATCTTGTTTCCAATTCCAAATTTCTTTCTTCCTTCATTGTTTTGTTTT
GTAGTAAATTGAGTTTGAGTTGAGAACATATTCATATTTGAGTTGAGTACTAAGCATGG-
GATCGCCATATTGCCGTTCCTCTTACCGTTAGGTCCTACGACAGGTAACCAATTGACTGGTGCTTGTACTCTTAGCGCATAA
GAGCCTAACCTGAAGTGTAGTATTACGAACTACAGTAACGGGTCCCGATTTTAATCCG-
TAAAACAGAGACCAACCAAACGGGAGCTATTCATTGTGCCAGACTGTCTGTTGAATAGTAAACATAAGTCTCCAGGCTAT
AGATCAGTATCCAATCTTATTGCACATAATACATGTCTAACTTTGCAATAAATGTGATA-
CATCATCTCCAACTCTCAAAGACTTTCGAAGATTCCTTTTCAAGTCTAAGAGGTCCAAGTCGTCACCAGGCTCGCTTACGA
GATCTTCATCTCGCAAAACGGGCTTATAATCAACCGTATCATATGAAGTTAAATCTCTAATT-
GTACGAGGGCGAGGATTTCGTGGCCTTGAAACATTCGTTTCTACAGGATCATATCGAGGAATAGCTTGCATAGTATTTTGC
TTAGGTCTTGTAGCAACTGTGTCTGCAGGATTTTGATCACGAACTACAACAGGCGC-
TATAGTAGGTTGTGTTAGTGGTGTTGATGAATAATCATGAAGTCCGTCAGTAAAATAATCTTTAGAAGGAACCTGAATAAT
ACTTGTCTTATTATCTGAATAGGTTGAATAAGGAGTTAAAAAGTGAG-
CATTATAAGGTAAAAACGGATCAGCAAATTGGAAATCGTCCCCTGCTCGGTATTCAAGTTCAAATGTTATTGTGAATTTT

GTACAGAAGACTCCAATTTACCGCGAAGGCCAAACAAAA-
TATAATTATCTCCATGAGGTTCATTATGAATATTTCGAAGACGTTGGACTCGAGTTGTTCCATTAACATCACAAGTCATAAA
ATTTTTTGCAGGACGAGAACGATTACCAATCCAATTAAATTGATCATAAAATGGAACAGGG-
TATTCAAAGGGAACTTCAACCTCAAAATGACGAAACATTGCCGTATCACCCATAACATACGAATTTATCATAGATTCTCGG
TAAGACCGATCTTCTCTTTGAATTCCAGGTGTAGTGTCCTCAGTAGGATAAATACCGACAAC-
GGATGGGGAATTACGAACTACAGATGAAAATATATATCCTTGTGTTGTGAATCCTGCTACTGTACGAATTCTATAATGCAT
AGTTCCTCGCCATTTCCTAAACATAGTGGCTAATGAAGCTAAAATTGGTGGTG-
CATCATATTGAGTAATATAAACAGCTTGTTGATCTGGGTCAATTTGTGTATAAGTTTTACCAGCATGTGTTACGTTACGCAT
GTTATTAAAAGCATAGCACTGATACGTATCCATTGTTGTTAGCTTTCTATTCTGAATACT-
AGGTTGTTTCGAGGTAGGATCAAGGGTTGTTTCACAAGAAGAATCTTTGTATTGATAAAATAACGTAGGGATAAATGGTCC
ATTTCGAATAGCAAAAATATAATCCTGATCATTTCCAG-
TAAAAGGCAAATTTAAAGTGACTCTAATACCCATGGGCTGCCATTGATGTATAAGATGATCAAAATCAAAAGGCCTAGGA
ATATTGCATTGTGGAATTTTAGATTGCTCAGGGAGTTTATGAGACTCGCTGGGCAATT-
GAACATTTTGTTCTGGAGTTACTTGCGGTTCTTCGTTACTCATGGTGCTGTATAAAAGGGTGATTCTTTAACGCATAATGAA
TATGTAGATTTTGAATCAGTTGGAATCATAAATTCAGTATTTTTAAAAGCTCGTTCAA-
TAATTATAGTATAACTATCCGGAAATATACTGCCAGGCGCTATAGTTTCAGCGACCTCCAATGAAATTGCTCCCATTGAAA
AATCGATCCAAGGTGTTAACGTAGTGGCATAACTAACTCTATCTTGTGCAGTTGAAGGAG-
TTATACTTTTAAGGCCAGGAAATTTAGTAGGACGGGCCCTAATAGGTAATGAAGCTGAAATATCAAAAGTAAAATGATTG
GAATCAGATAAATCCCATTCCTTTAATATTGATCGATAAGTGCTGTCTTTAATATTGC-
TACTATTGGCTGGCCTACCATTAAAAACTAAGAAGTTATCTTGTCTGTAACGAACTAAAAGCTTTCCAGTAACACGCGGTG
GTTTAATAATAGACAAACGATAAGAAACAATTCCTTTCCACCAAATAGATGAA-
GCAAAAGGAATTCTTTCCCAATTTGGATAAGTACGAAAGACTGTATTACCGTTATTAGGATGCAAAACATCGGTCGAAAC
TATAATGGATCCTACCGCATTAGTAGTACTAACTTGTT-
GTGTGCTTAAAATTTTATACTGTTCAACCATCCAAGAGAATTGTGGTGGTAAAGATGAAATGTTCTCTAATGGTTCAAATG
GTATTCCAGTATGTTCAAGTAGCCCAGTGTTTGTCTGAGGTACAGGGTTCTGAGGCAAACCA-
TAATTTCGAACTGGATTATGGGTATCTGCATTAATTTCTTCATTTAATGACATTATATATTAGATTGAATATTTGTTTGACCT
GATAAATTAGCAGTTGTACCAGAATTTACAGAGCCAGTATCTTGTGGATTAAATCGAC-
CTTCAAATGAATAGCCTGTTTTCAAATCATTATATGTATCTTTGGGTGCCGAGTCTTGAATAGCATTAGCAAGTGAACCTCC
CAACCACGCGCCAACAGGACCAAAAAGTCCACCAATTTGAGCGCCAGAAGCAGTAACGTTAG-
CATGAGCGGTATCAATTTCCTTTAGAAGTTGTGCTTGATGAGTTGATTGTGATCCTTGAACTTTAGTATTTGCTACATAATCA
GATGCTATAGTTGATTTATTTCCAGCATCAATAGCACTAGCAGTAGCAGCACCAGCGGCAG-
CATTAATTGCAGCAGCAATACCAGCAGGTCCAGTTAAGGAGGCTACTGATAAAGCAGCATTGCCAACACCTGTAACAGCG
CTAGCAACTTTTGCAACGTCCTTTGATTGAGCTAATTTTGTAGGGCCTTT-
GGTTTCTGCTGCTGCCAGAGGGGCAATAGGGACTCTAGATTGGGGCGCGGAACTAGTCGACGGTATCGCAGGCGTCGTTT
GGCTAAGTGCATTTTGCGCAGATTGTTTTTCAATTCTTGACCACTGTGAACGAACTCCTGA-
TAAGTCCGATTCACGGTTGGTCCAATATGGAATAGGATCATTAGTAGAATTCTGGCTAATGGAAAATATACCTTCTGAGTG
AGGATCCCAGTGATTGACCTGATCATACCACGATATTCCTGCCATTTCGGGAGATTGG-
TAAATTGGATTTGTAGTCCAGGTAACATTAGGGCCTGATAAAGAAGTTGAATTTGATGATTTTACAAAATTCATATAATGTT
ATCTTGAGGAACATAACCAGAATATTCAACATCGGTAAGATGTGACCAAATTCTTACTG-
TAGCGTGTGGGGTAACACCGGAAGCAGTTGTCAACGGAATTGTATTAACTAAATAAACAAAGCCCATATCATAAAGCGG
GTCGTTAGGATCTTGATATTTATCGCCAAATACATAATTAGGATTAAAAGGTTTAATTCGTG-
TAAAAGATGATTTAAATGGTGAAAGCCAATCAAATGTACACTCTACATCTTGGTTTTCTCCCAACATTACATGAACATGAG

GCAACTGATTCATGGATGTTAAACTAAATAAAGAATGATTTGTTATATTCGCTGCAG-
TAACAAGGCCATTTTGTCCAGGAGTATCATTTTTAGTGATCTGTGTATCAACATAAGAAATAGGAGCTGAAGTTAAATAAG
GAATAGCATCAAGTGGAACGTTAGCATAAGCAATAGTCATCATTCCAACTTCCTGAAAATTT-
GATCGAAATTCAAAAGTTAAATGCCATTTCCACGACTTTAAAAAGAATAAATCTGATAAATTTCTAAAATGCAATTTTTGA
ATATTATTCCAAGAATTTTGAAATAGAAATAAGGGAGTCGTACCGGTTCCATTTGTGG-
GAATTGCAATGGTCGTCATAAGTTGTTTCTGCGAGACTAACTGCTCAAAAGCCCATTTAGTAGCAATGACCGATGACCTGT
CATCAAAAGCTGATCCAGGGGCTGATGGTATATTAAGCATATTAGATTCCATTGGCAAGGG-
TATAGTACCGTGGCCAATTTCTTTTGCTCGACTCTTGTCGATGCTTTCTTCAGTTGGTTGTATTATATTCATGATAATTTAAAG
ATGTTATTAAAATAATGGGCAT-
ATCAACATAATTTCTTCGTCCCATTAAATTAATTTTCATTCATACATGCATACAAAAGTTGAATTTTAGAATTTCCATTCTTT
AAAATAATTGGAGATGTAAGGGTCCGTAGACACGCGATAGAGAGCTCCAA-
GAACACATCTTCGTGAAGAATGTGAAGCATACATAGGGGGTGACCCTATG
>TRINITY_DN93_c0_g1_i7
TTTTTTTTTTTTTTAAAACTAAATAATAATAATAATAATAATAATAATTATAATAA-
TAATTAATAATTAATAAAATGGAAAAGAAAAACACGGGTAGCACCTTCATGCCCGATGGTGGCTTCACTAGAATCCAATG
CATCCGTGCTTTTAGAACTTACGAAACAAGTTCTAAGCAACAAATAGGTTA-
GAGGTAATTTAGTTTAGACGCCTCTGCCTTTAGATGTTAAAACAAATTGCCAAAACTTAAACTATGCCCAGCCACGGGGGA
TCTAAATACACTGTTACATGTCTAAGAATTAACTTAATAAAATAGCTGTTCCACTATCCG-
CAAGCGGATCAGTAACTAGCATCCAGCCTCCGAGAGGGTTTCAGCTAGTCTCACAATGCGACGATCGGAACAGACCTACC
TTATATCCCGTTAAATTACATTACGGGTATAATACTAACTTAAAATAAAATGAT-
TATTCTGAGTAATATTTTTGGATTTTATCTTTTTCGAAATCCTCCCATCGATAGTCAAAGGGCTCAAGATTGCACTCACGCA
ACCATTGGTTAACTTCGTCCCTTACCTCATTATATTTATTGGGGCCATGACCGTGGAT-
TAAACGAAAAGAATCAAAGAGATTTGAGCGAAATTGATCATAAGGATGTTCTTTCGCACGGACCCAGTACATAAGATCGT
AACATACTGATATGTCCATCAAGCGATCCACTCGAGATGGATTAATATAGTTGAAACCTGAT-
TTTAAGAAAGTACATTCAAAAATGTTTTTACTTTTGGGCATCTCTTTTGTTTTTGAAGCAGTTGTGACTGGATATCCATGTTC
AATGTACATACGAGAAATCGTATTTCCATTGAACCATGGAATTATATGTTCAG-
CAACTGACATAATGACATCATCCCCGTAAAAGACGGGTGACGTGTAATAAAAGAAATCATGAATATTTGCATATTGTTGG
TTATCAGTTAAATCAGCAATATAAAGGTAAAAATAATACATGAGTAATAAATGAAC-
GAGGGTGTTGACCTCAGCAGTTCCAGGAAATCCTGAAATCAACCCCCTGCATTTTTGATAAACCATATCTTCAAATTGGAC
ATGGCCAAATAAAACCTCGGTTAATAATGAGTAAATAACTTTAGCGGTGGGA-
GAATCAAATGAAACGCGGAGGATAATACATAACATATCAGCTACGGATAATAATAATTCAGGAGGCATATGGCCATCCC
AGTTACTTACATCAAAATCTAAGCAATTAGGGTGTTTATTTAAATAATGAAAGAGGCGAG-
TCCAATCAGGACCTTCAGGATTAATCCCGGGACCAAAGGGAAAATCTCCTCGAGCGGCACGATGTAAAGATGCCATCAA
ATCTAGTGTCACACGACGCCATGAAAATATCATTTCCATGTTCATACACGTGACAGATCTT-
GTTTTCGGGAGAGTCCCAGTGACTGGATCACCAAGTGCTTTATAATACGGGCGAAGTTCATCTTTTGGAAAGTCATATGAT
GAATGTTTCGGAATAACGCCTTGTTGTAATTTTGCAAAGGTGGCTTCAAATGAT-
TTTACAAATTCAGGATCATTAACTTTACACCGTCCTTCATCAGTAATCTCGACATAGGCTTTCTTGCCTTTTGGCTTTCCATG
ATATTTATCCAATACATATGGAAGACCAGCAGACTTACTGCAATTCACAGGATTGCTAC-
CTGGCTCACGAATACCTGTAACACAGGTTTCGAAATCCAGGTTACAATCAAAGGTTTGTTTATCTAACCTGTCGCGCAGCC
AATACGCCATATCTTCGGTGGCTCGATTCAAAATAGCCAAGTCAAATGACCCAACTTT-
GCCTGTGCCATGTTTATTAACAGAATGTTGCATAGGATGTGCATGAACTAAAAGACGATGATCGTGAGGATTCAAAGCAG

CAGGAACCCGAGGGGATGTTTTCCCTGATTTGTCCATCTCGTGGGCTATAA-
TAGTTTTCTTAAAAGCAGTGCGGGCCACCATGCCAACGACGTGTTCAGCCGGAACTGAACCACAAATGTTGACATGGGAA
TCCACAATGCCCTCAACTTTAGACGCTGTAGGTTCACAAAGAATAGGGCCTTCTTGAG-
TTATAACAGGACGTTTAACTTGTTTTTCAACCTGTTTCATTAATTCAGAAAGAAGCTCTTGAGTTACTACTTGGTAAACTATC
GTCTGTTTATAGTAATCTGAAACTTTCCAGGCTTGAATTCCAACTATGGAGCGATGCCCTG-
GACGCTTGCTTGCTGACATTAGCATTGAACCACTTTTACCTCCCACAGTAACTCCTTCAACCATAATTGCCATCGACATTTC
TCCTCGTGCTCCATCGTTGGCAGTCATAGCAACCTTCTTATGGGGAACGCAGCGGTCAC-
GAATTTCGACTGATTGTTGATTTTCATAATTTGTAAGAGACCAGAGCTCACCGATATTCTCGGATTGCTCAAAATCATCATT
AGTAATAAATTGATTAGTTATATCAGAGGCCATAGGTAAATGACGAGAAAATATAATGGCAA-
GATCATTGCCAGGAGATTCAAAAACATTATCACGAGAAATTTCAATCTCCCATGCTTTATCAGAATTAACAGTAGGTGTAT
AAGACAATATAGTAGGTCCTTTAATGTGCATAGTAGTATGGGAATTTAAAATTAAAAATT-
GCTTTGTATGAATTCCATGAGCTTTCACATCAAGGCCATCTTCATTAACAATATGGAAAAATTTAATATGCTTATCTAAATA
TTTTTGGGCTAAAATTTGTTGCGTATTGGATGGATCAAAAATTCCTGCTTGTGGTCTAC-
CTCTGAACATCAAGTTTGATTTTATGTTTGAGCGATGCATAAATTTGGAAGTGGGTTCAGATGAGCCGAAGAACAGTTTTC
CCACTTGTTTAAGTAAATAAACCGTTCCCGCAAATGCTGCAATTTTTAAAAATGAAGAGA-
CAATTGTAGAAAGCTTATTCCAGACCCAACCTACAGCGCGGTAAACATGAGCTAGGGTAACTCTTTTCACTGTATGGACTA
TCGATAAAATTCGCTCTCTAAGATGTTCTCGTGATAAAGAATACAT-
ATGTTCATATTGCCATTTGGCAGCTGAAACCATATCACGCTGTTCCTCAGCTGAGAGCAATGAAAATAAATTTGCTGTATA
AGTAAATGATTGATTTATAGAGCAGAAAAATGCTATATCTAAAGGTATATGA-
TAAGTTTTCTGAATGTTATTCAAATTTTTCTTAATCGCTATAGAATTTGTAATATGAAAATCAAGATCTGATACATCCATATG
CCAATCGCCATCAACATAGCTAAGGCGACGAAGAAACTCAATGGATATTCGTGAATTTCCTT-
GCTCAATATGTTCATCAGAATAAGGTTTACGATTAGGGTAATAAAATTTGTGCTCTGAATTTACTTGTTTGAAAAAGGACAT
CATTTTCGGTTTATCCCACATAAATCGCGTTTTATAATTTTGATAAGATGATTCCATTACTT-
GATCTGATTGCAAATAATTATAAAAATTTTCTCCAGGGAAAGTTCCTGATTCGGAACAACCCCAATTAGTATAATAGGACC
AAATTGGTATAGCTGGATAAAGATTGCCTTCTGAATTATGAACTAATTTGACAAC-
CATCTGCTCTTTAATCAAGGGATCAATAATACGTTTATTTAATTTTTGCATAATACGTTGACGTCTAGATGTTTCAGGGCCA
GGATTACCAGGAACAATATCTTGATAGGCTTCTTTAGGAAAAGGATTTGGTGCCTTAC-
CATCTAAATATGAAAAGTAGCGATAATCTGAAATTGTGAAATGCTCTTTCATGGCACACTTATGCATCAGCAATAAGACTA
AATCGCGTTCGGGTCCTGTCAAATCATGAGATCGAATGTGCTCAGTCAG-
TTTTTCAAGTTTAATCTCTTCATCTGTTAAAGTAAGACCCAAAAATTCATTTGGGTCACCATAACTAGATTCCCAATCATCA
TCATCGACGGTACCGGCAGTTGGCATACCTTCTGTAGAATCTAAAAACTCCTGAGTTATAC-
CATCGATGTCGAGATCACACATGTCTGGAAGTACTTGATCTAAAGTCTGAGCATAAACACGCTTTCCAAGAGGATCATCC
ATGCCATATTGAAGAGAAGCATCTAAAAATTTATCAGAAATAAGTTTAAATGTGGTT-
GATGCAGCCACACCGTCTGAGATATCATCTGATTGAACAAATATATTGTCAATTTCAGCGAAGCATTCCATAACATGTTCA
TATTTTTCCTTAACTGTTAGTTTTTGCTCTCCTGACCTAAGATGTTGATAACTAACCGC-
TACATTTTCAACAAAAGTATCAAATGTCCAGTTAGTACAAGGAACTGTCATTCCTTTCAAAGGGCGAGCATCGGGACCGA
AGAAAAAGTCTTCATTAAATTTTAGAGTTTCATT-
AGCTGTGCGAAGTTTCTTAATTAAATTATGCTTCCACTCCATCTCATCAGAAGTTGTACTCTGATATTGCTCTTCACCAGGAT
TGATAACAGGTTTCATTAAAGCAAATTTAAGATGAGGAAAATCAAACGAATTTTGATTCGGA-
TAAAATTTCTCAAAAAGTTGTTTTGAAAATTTACCATCTGCTTTAACACGAGGGTCCATTTTGACTTCACACAATACATGAC

GACGTCTGTGAACAGCCTCCATGCAAAATATGTCTTTTCCGATTGGATAGGGAACATTAG-
TAGAAGAGATTAAAATATCCGAATCCAAATGAACTCCTTTATCCTCTAAGTGAGCCATAGGAAGGGGGACTGGTGTATTA
GTAATAAGTCCTATAACTAAAGATAAATGTTT
>TRINITY_DN93_c0_g1_i2
TTTTTTTTTTTTTTAAAACTAAATAATAATAATAATAATAATAATAATTATAATAA-
TAATTAATAATTAATAAAATGGAAAAGAAAAACACGGGTAGCACCTTCATGCCCGATGGTGGCTTCACTAGAATCCAATG
CATCCGTGCTTTTAGAACTTACGAAACAAGTTCTAAGCAACAAATAGGTTA-
GAGGTAATTTAGTTTAGACGCCTCTGCCTTTAGATGTTAAAACAAATTGCCAAAACTTAAACTATGCCCAGCCACGGGGGA
TCTAAATACACTGTTACATGTCTAAGAATTAACTTAATAAAATAGCTGTTCCACTATCCG-
CAAGCGGATCAGTAACTAGCATCCAGCCTCCGAGAGGGTTTCAGCTAGTCTCACAATGCGACGATCGGAACAGACCTACC
TTATATCCCGTTAAATTACATTACGGGTATAATACTAACTTAAAAATAAAA-
TAATTATTCTGAATAGTATTTTTGAATTTTATCTTTTTCGAAATCCTCCCATCGATAGTCAAAGGGCTCAAGATTGCACTCAC
GCAACCATTGGTTAACTTCGTCCCTTACCTCATTATATTTATTGGGGCCATGACCGTGGAT-
TAAACGAAAAGAATCAAAGAGATTTGAACGGAATTGATCGTAAGGGTGTTCTTTTGCTCGCACCCAATACATTAAATCAT
AGCAAACTGAAATATCCATCAAGCGATCCACTCGAGATGGATTAATATAGTTGAAACCTGAT-
TTTAAGAAAGTACATTCAAAAATGTTTTTACTTTTGGGCATCTCTTTTGTTTTTGAAGCAGTTGTGACTGGATATCCATGTTC
AATGTACATACGAGAAATCGTATTTCCATTGAACCATGGAATTATATGTTCAG-
CAACTGACATAATGACATCATCCCCGTAAAAGACGGGTGACGTGTAATAAAAGAAATCATGAATATTTGCATATTGTTGG
TTATCAGTTAAATCAGCAATATAAAGGTAAAAATAATACATGAGTAATAAATGAAC-
GAGGGTGTTGACCTCAGCAGTTCCAGGAAATCCTGAAATCAACCCCCTGCATTTTTGATAAACCATATCTTCAAATTGGAC
ATGGCCAAATAAAACCTCGGTTAATAATGAGTAAATAACTTTAGCGGTGGGA-
GAATCAAATGAAACGCGGAGGATAATACATAACATATCAGCTACGGATAATAATAATTCAGGAGGCATATGGCCATCCC
AGTTACTTACATCAAAATCTAAGCAATTAGGGTGTTTATTTAAATAATGAAAGAGGCGAG-
TCCAATCAGGACCTTCAGGATTAATCCCGGGACCAAAGGGAAAATCTCCTCGAGCGGCACGATGTAAAGATGCCATCAA
ATCTAGTGTCACACGACGCCATGAAAATATCATTTCCATGTTCATACATGTGACAGATCTT-
GTTTTAGGGAGAGTTCCTGCAACTGGATCGCCAAGTGCTTTATAATATGGACGAAGCTCGTCTTTTGGAAAATCATATGAT
GAATGCTTTGGAATAATGCCTTGTTGCAATTTTGAAAAAGTGGCTTCAAATGATTTAA-
TAAACTCGGGGTCATTCACTTTGCATTGGCCTTCTTCAGTAATTTCGACATAGGCTTTCTTGCCTTTTGG
>TRINITY_DN93_c0_g1_i8
TTTTTTTTTTTTTTAAAACTAAATAATAATAATAATAATAATAATAATTATAATAA-
TAATTAATAATTAATAAAATGGAAAAGAAAAACACGGGTAGCACCTTCATGCCCGATGGTGGCTTCACTAGAATCCAATG
CATCCGTGCTTTTAGAACTTACGAAACAAGTTCTAAGCAACAAATAGGTTA-
GAGGTAATTTAGTTTAGACGCCTCTGCCTTTAGATGTTAAAACAAATTGCCAAAACTTAAACTATGCCCAGCCACGGGGGA
TCTAAATACACTGTTACATGTCTAAGAATTAACTTAATAAAATAGCTGTTCCACTATCCG-
CAAGCGGATCAGTAACTAGCATCCAGCCTCCGAGAGGGTTTCAGCTAGTCTCACAATGCGACGATCGGAACAGACCTACC
TTATATCCCGTTAAATTACATTACGGGTATAATACTAACTTAAAATAAAATGAT-
TATTCTGAGTAATATTTTTGGATTTTATCTTTTTCGAAATCCTCCCATCGATAGTCAAAGGGCTCAAGATTGCACTCACGCA
ACCATTGGTTAACTTCGTCCCTTACCTCATTATATTTATTGGGGCCATGACCGTGGAT-
TAAACGAAAAGAATCAAAGAGATTTGAGCGAAATTGATCATAAGGATGTTCTTTCGCACGGACCCAGTACATAAGATCGT
AACATACTGATATGTCCATCAAGCGATCCACTCGAGATGGATTAATATAGTTGAAACCTGAT-
TTTAAGAAAGTACATTCAAAAATGTTTTTACTTTTGGGCATCTCTTTTGTTTTTGAAGCAGTTGTGACTGGATATCCATGTTC

AATGTACATACGAGAAATCGTATTTCCATTGAACCATGGAATTATATGTTCAG-
CAACTGACATAATGACATCATCCCCGTAAAAGACGGGTGACGTGTAATAAAAGAAATCATGAATATTTGCATATTGTTGG
TTATCAGTTAAATCAGCAATATAAAGGTAAAAATAATACATGAGTAATAAATGAAC-
GAGGGTGTTGACCTCAGCAGTTCCAGGAAATCCTGAAATCAACCCCCTGCATTTTTGATAAACCATATCTTCAAATTGGAC
ATGGCCAAATAAAACCTCGGTTAATAATGAGTAAATAACTTTAGCGGTAGGAGAATCAAAA-
GAAACACGAAGGATTATACATAACATGTCAGCTACGGATAATAATAATTCGGGTGGCATATGGCCATCCCAATTACTTAC
ATCAAAGTCTAAACAATTAGGGTGTTTATTCAAGTAATGAAATAGGCGAG-
TCCAATCAGGGCCCTCAGGATTAATCCCAGGACCAAAAGGAAAATCTCCACGAGCAGCGCGATGTAAAGACGCCATCAA
ATCTAGAGTCACACGACGCCATGAAAATATCATTTCCATGTTCATACATGTGACAGATCTT-
GTTTTAGGGAGAGTTCCTGCAACTGGATCGCCAAGTGCTTTATAATATGGACGAAGCTCGTCTTTTGGAAAATCATATGAT
GAATGCTTTGGAATAATGCCTTGTTGCAATTTTGAAAAAGTGGCTTCAAATGATTTAA-
TAAACTCGGGGTCATTCACTTTGCATTGGCCTTCTTCAGTAATTTCGACATAGGCTTTCTTGCCTTTTGGTTTTCCATGATATT
TATCTAATACATATGGGAGGCCAGCTGACTTACTACAATTGACTGGAT-
TACTGCCTGGTTCTCGAATACCCGTAACACAGGTTTCAAAATCCAAATTGCAATCAAAAGATTGCTTGTCTAATCTGTCAC
GCAGCCAATATGCCATATCTTCAGTGGCTCGGTTCAAAATTGCTAGATCAAAAGATCCAAC-
CTTTCCTGTGCCATGCTTATTGACTGAATGTTGCATAGGATGAGCATGAACTAAAAGCCGATGATCATGAGGATTCAAAGC
AGCTGGAACACGAGGAGATGTTTTTCCTGACTTGTCCATCTCATGGGCAATAATT-
GTTTTCTTGAAGGCAGTTCGAGCTACCATACCAACAACATGTTCAGCCGGAACCGAACCACAAATGTTGATGTGAGAGTC
AACAATACCTTCCACTTTTGAAGATGTGGGTTCACATAAAATGGGGCCTTCTT-
GGGTTATAACAGGACGTTTTACTTGTTTTTCAACCTGTTGCATTAATTCGGAAAGAAGTTCTTGAGTAACTACTTGATAAAC
TATGGTCTGCTTATAATAATCTGATACTTTCCAGGCTTGAATACCTACTATTGAAC-
GATGTCCTGGACGCTTACTAGCTGACATTAGCATTGAACCACTCTTTCCTCCAACTGTCACTCCCTCAACCATGATAGCCAT
TGACATTTCTCCTCTTGCTCCATCATTAGCAGTCATGGTGACTTTCTTATGGGGGACACAAC-
GATCACGAATCTCAACAGATTGCTGATTTTCATAGTTTGTAAGAGACCAAAGTTCGCCAATATTTTCAGATTGTTCGAAATC
CTCATTAGTAATAAATTGATTAGTTATATCAGAGGCCATAGGTAAGTGACGAGAAAAAA-
TAATTGCAAGATCATTTCCAGGGGATTCAAAAACATTATCACGGGATATCTCGATTTCCCATGCTTTATCAGAATTAACTGT
AGGGGTATAAGATAATATAGTTGGGCCTTTGATGTGGGTTGTAGTATGTGAATTT
>TRINITY_DN93_c0_g1_i9
TTTTTTTTTTTTTTAAAACTAAATAATAATAATAATAATAATAATAATTATAATAA-
TAATTAATAATTAATAAAATGGAAAAGAAAAACACGGGTAGCACCTTCATGCCCGATGGTGGCTTCACTAGAATCCAATG
CATCCGTGCTTTTAGAACTTACGAAACAAGTTCTAAGCAACAAATAGGTTA-
GAGGTAATTTAGTTTAGACGCCTCTGCCTTTAGATGTTAAAACAAATTGCCAAAACTTAAACTATGCCCAGCCACGGGGGA
TCTAAATACACTGTTACATGTCTAAGAATTAACTTAATAAAATAGCTGTTCCACTATCCG-
CAAGCGGATCAGTAACTAGCATCCAGCCTCCGAGAGGGTTTCAGCTAGTCTCACAATGCGACGATCGGAACAGACCTACC
TTATATCCCGTTAAATTACATTACGGGTATAATACTAACTTAAAATAAAATGAT-
TATTCTGAGTAATATTTTTGGATTTTATCTTTTTCGAAATCCTCCCATCGATAGTCAAAGGGCTCAAGATTGCACTCACGCA
ACCATTGGTTAACTTCGTCCCTTACCTCATTATATTTATTGGGGCCATGACCGTGGAT-
TAAACGAAAAGAATCAAAGAGATTTGAACGGAATTGATCGTAAGGGTGTTCTTTTGCTCGCACCCAATACATTAAATCAT
AGCAAACTGAAATATCCATCAAGCGATCCACTCGAGATGGATTTATATAGTTGAAACCGGAT-
TTTAAAAAAGTACATTCGAAAATATTTTTACTTTTAGGCATCTCTTTGGTTTTTGAAGCAGTTGTGACAGGATATCCATGCTC
AATGTACATGCGAGAAATCGTGTTTCCATTAAACCATGGTATAATATGTTCAG-
CAACTGACATAATAACATCATCCCCGTAGAAGACAGGTGATGTATAATAAAAGAAATCATGAATATTTGCATATTGTTGG

TTTTCTGTTAAATCGGCAATATAAAGATAAAAATAATACATGAGTAATAAGTGAACGAGAG-
TGTTGACTTCGGCAGTTCCAGGAAATCCCGAAATCAACCCTCTACATTTTTGATAAACCATATCTTCGAATTGGACATGGC
CAAACAAAACTTCGGTTAATAATGAGTAAATAACTTTAGCGGTAGGAGAATCAAAA-
GAAACACGAAGGATTATACATAACATGTCAGCTACGGATAATAATAATTCGGGTGGCATATGGCCATCCCAATTACTTAC
ATCAAAGTCTAAACAATTAGGGTGTTTATTCAAGTAATGAAATAGGCGAG-
TCCAATCAGGGCCCTCAGGATTAATCCCAGGACCAAAAGGAAAATCTCCACGAGCAGCGCGATGTAAAGACGCCATCAA
ATCTAGAGTCACACGACGCCATGAAAATATCATTTCCATGTTCATACA
>TRINITY_DN15442_c0_g1_i1
CATTTGCTGTACGGAGTTTCTTAATTAAATTATGCTTCCACTCCATCTCATCAGAAGTT-
GTGCTCTGATATTGTTCTTCTCCAGGATTAATAACAGGCTTCATTAACGCAAATTTAAGATGAGGAAAATCAAACGAATTC
TGATTGGGATAAAATTTCTCAAAAAGTTGCTTCGAAAATTTGCCATCTGCTTTTACACGGG-
GATCCATTTTAACTTCACATAAAACATGACGGCGTCTATGAACGGCTTCCATACAAAAAATGTCTTTCCCAATTGGATAAG
GGACATTTGTAGATGAAATTAAAA-
TATCTGAATCTAAATGAACTCCCTTATCTTCTAAATGTGCCATGGGGAGTGGAACAGGAGTATTAGTAATAAGTCCTATAA
CTAAAGATAAATGTTTGGGTTCATTATATCTAAATAGATCATCGATAATCATAAAAGTTT-
GACCATTATAACCATCAAAATGATCTACATTGGGATTATAAGTCCAATGAGTTATATTTTTGTCTTCGCGATAAATTTTCTT
AGATAAAGATGTAATAAATTTTTCAGTTAAAGTAGATTTTCCAATTCCAGGTTCACCAACAAATT
>TRINITY_DN15442_c0_g1_i2
CATTTGCTGTACGGAGTTTCTTAATTAAATTATGCTTCCACTCCATCTCATCAGAAGTT-
GTGCTCTGATATTGTTCTTCTCCAGGATTAATAACAGGCTTCATTAACGCAAATTTAAGATGAGGAAAATCAAACGAATTC
TGATTGGGATAAAATTTCTCAAAAAGTTGCTTCGAAAATTTGCCATCTGCTTTTACACGGG-
GATCCATTTTAACTTCACATAATACATGGCGACGTCTATGAACAGCTTCCATGCAAAATATGTCTTTTCCAATTGGGTAGGG
AACATTAGTAGAAGAAATTAAAA-
TATCTGAATCTAAGTGAACTCCTTTATCTTCTAAATGGGCCATAGGAAGAGGAACTGGTGTATTAGTAATAAGTCCTATAA
CTAAAGATAAATGTTTGGGTTCATTATATCTAAATAGATCATCGATAATCATAAAAGTTT-
GACCATTATAACCATCAAAATGATCTACATTGGGATTATAAGTCCAATGAGTTATATTTTTGTCTTCGCGATAAATTTTCTT
AGATAAAGATGTAATAAATTTTTCAGTTAAAGTAGATTTTCCAATTCCAGGTTCACCAACAAATT
>TRINITY_DN15442_c0_g1_i3
CATTTGCTGTACGGAGTTTCTTAATTAAATTATGCTTCCACTCCATCTCATCAGAAGTT-
GTGCTCTGATATTGTTCTTCTCCAGGATTAATAACAGGCTTCATTAACGCAAATTTAAGATGAGGAAAATCAAACGAATTC
TGATTGGGATAAAATTTCTCAAAAAGTTGCTTCGAAAATTTGCCATCTGCTTTTACACGGG-
GATCCATTTTAACTTCACATAATACATGGCGACGTCTATGAACAGCTTCCATGCAAAATATGTCTTTTCCAATTGGGTAGGG
AACATTAGTAGAAGAAATTAAAA-
TATCTGAATCTAAGTGAACTCCTTTATCTTCTAAATGGGCCATAGGAAGAGGAACTGGTGTATTAGTAATAAGCCCTATAA
CTAAAGATAAATGTTTAGGCTCATTGTATCTAAATAAATCATCGATAATCATAAAAGTTT-
GACCATTATAACCATCAAAATGATCTACATTGGGATTATAAGTCCAATGAGTTATATTTTTGTCTTCGCGATAAATTTTCTT
AGATAAAGATGTAATAAATTTTTCAGTTAAAGTAGATTTTCCAATTCCAGGTTCACCAACAAATT
>TRINITY_DN17024_c0_g1_i1
TTAGAGGGAACTTGAATAATGCTTGGACTATTGTCGGAATAAGTTGAA-
TAAGGGGTTAAAAAGTGAGCATTATAAGGTAAAAATGGATCAGCAAATTGAAAATCATCTCCCGCTCGATATTCAAGCTC
AAATGTAATTTGCGAATTTTGTACGGAAGATTCCAATTTTCCAC-
GGAGGCCAAACAATATATAATTATCTCCATGAGGCTCATTGTGAATATTTCGAAGACGCTGAACTCGAGTTGTCCCATTAA

TGTCACAAGTCATAAAATTTTTTGCAGGGCGAGTACGATTTCCAATCCAATTAAATT-
GATCATAAAATGGAACAGGATATTCAAAGGGAACCTCAACTTCAAAATGACGAAACATTGCTGTGTCACCCATAACGTAC
GAATTTATCATTGACTCTCGATATGACCGATCCTCCCTTTGAATTCCAGGGGTAG-
TATCCTCAGTAGGCTAAATGCCGACAACAGATGGAGAATTACGAACTACAGATGAAAATATGTATCCTTGCGTTGTGA
>TRINITY_DN3718_c0_g1_i1
TGTGAATTCCATGAGCTTTAACATCAAGGCCATCCTCATTAACGATGTGGAAAAACTTAA-
TATGCTTATCTAAATATTTTTGGGCTAAAATTTGTTGAGTGTTGGATGGATCAAAAATTCCTGCTTGAGGTCTACCTCTAAA
CATCAAATTTGATTTGATATTAGAGCGATGCATAAATTTAGAAGTAGGTTCG-
GATGAGCCAAAGAAAAGTTTTCCCACTTGCTTAAGTAAATAAACTGTTCCAGCAAATGCTGCTATTTTTAAAAATGAAGAT
ACAATTGTAGAGAGCTTGTTCCAAACCCATCCTACAGCACGGTAGACATGGGCGAGAG-
TAACTCTTTTCACAGTATGAACTATTGATAAAATTCGCTCTCTAAGATGTTCTCGTGATAAAGAATACATATGTTCGTATTG
CCATTTAGCAGCTGAAACCATATCGCGTTGTTCTTCAGCTGAAAGCAATGAAAATAAATTT-
GCCGTATAAGTAAATGACTGATTTATAGAGCAGAAAAAAGCTATATCTAAAGGTATATGATAAGTTTTCTGAATGTTATTC
AAATTTTTCTTAATTGCTATAGAATTCGTAATATGAAAATCAAGATCTGATA-
CATCCATGTGCCAATCACCATCAACATAACTGAGGCGACGAAGAAATTCAATGGATATTCGCGAATTTCCTTGCTCAATAT
GTTCATCAGAATAAGGCTTACGATTAGGATAATAAAATTTGTGCTCTGAATTTATTT-
GTTTAAAAAAAGACATCATCTTTGGTTTATCCCACATAAATCGCGTTTTATAATTTTGATAAGAGGACTCCATAACTTGATC
TGATTTTAAATAATTATAAAAATTCTCTCCTGGGAAGGTTCCTGACTCGGAACAACCCCAG-
TTAGTATAGTAAGACCAAATCGGGATAGCTGGGTAAAGATTGCCTTCAGTATTATGCACTAATTTAACAACCATTTGTTCTT
TCACTAAAGGATCAATAACGCGTTTATTTAATTTTTGCATAATACGCTGACGCCTA-
GATGTTTCAGGTCCAGGATTTCCAGGGACAATATCTTGATATGTTTCCTTAGGGAATGGATTTGGGGCCTTATTGTCTAAAT
ATGAAAAATAACGATAATCTGAGATAGTAAATTGCTCCTTCATAGCGCACTTATGCATTAA-
TAATAAAACTAAATCGCGCTCTGGGCCTGTTAAATCATGAGATCGAATATGCTCAGTTAGCTTTTCAAGCTTAATCTCTTCG
TCTGTTAAAGTAAGACCCAAAAGCTCATTAGGATCCCCGTAACTAGACTCCCAG-
TCATCATCATCGATGGTGCCTGCTGTTGGCATTCCTTCAGTGGAATCCAAAAATTCTTGTGTTATTCCATCGATATCAAGAT
CACACATATCTGGAAGTACTTGATCTAACGTTTGAGCATAAACACGCTTTCCAAGGG-
GATCATCCATACCATATTGAAGGGAAGCATCCAAAAATTTATCTGAAATAAGTTTAAATGTGGTGGAAGCAGCAACACCA
TCTGAAATGTCATCTGATTGCACAAAAA-
TATTATCAATTTCAGCGAAACATTCCATGACATGCTCGTACTTTTCTTTCACAGTGAGTTTTTGTTCACCTGATCTAAGATGT
TGATAACTAACTGCAACATTCTCAACAAAAGTATCAAATGTCCAGTTAGTGCATGGAACTGTCATTCCTTTCAAAGGACG
>TRINITY_DN3718_c0_g1_i2
AAAGGGTCAATAATACGTTTGTTTAATTTTTGCATAATACGTTGACGTCTA-
GATGTTTCCGGGCCAGGATTACCAGGAACAATGTCTTGATACGCTTCTTTAGGGAAAGGATTTGGTGCCTTGCCATCTAAA
TATGAAAAATAACGATAATCTGAGATAGTAAATTGCTCCTTCATAGCGCACTTATGCATTAA-
TAATAAAACTAAATCGCGCTCTGGGCCTGTTAAATCATGAGATCGAATATGCTCAGTTAGCTTTTCAAGCTTAATCTCTTCG
TCTGTTAAAGTAAGACCCAAAAGCTCATTAGGATCCCCGTAACTAGACTCCCAG-
TCATCATCATCGATGGTGCCTGCTGTTGGCATTCCTTCAGTGGAATCCAAAAATTCTTGTGTTATTCCATCGATATCAAGAT
CACACATATCTGGAAGTACTTGATCTAACGTTTGAGCATAAACACGCTTTCCAAGGG-
GATCATCCATACCATATTGAAGGGAAGCATCCAAAAATTTATCTGAAATAAGTTTAAATGTGGTGGAAGCAGCAACACCA
TCTGAAATGTCATCTGATTGCACAAAAA-
TATTATCAATTTCAGCGAAACATTCCATGACATGCTCGTACTTTTCTTTCACAGTGAGTTTTTGTTCACCTGATCTAAGATGT
TGATAACTAACTGCAACATTCTCAACAAAAGTATCAAATGTCCAGTTAGTGCATGGAACTGTCATTCCTTTCAAAGGACG

>TRINITY_DN3718_c0_g1_i3
TGTGAATTCCATGAGCTTTAACATCAAGGCCATCCTCATTAACGATGTGGAAAAACTTAA-
TATGCTTATCTAAATATTTTTGGGCTAAAATTTGTTGAGTGTTGGATGGATCAAAAATTCCTGCTTGAGGTCTACCTCTAAA
CATCAAATTTGATTTGATATTAGAGCGATGCATAAATTTAGAAGTAGGTTCG-
GATGAGCCAAAGAAAAGTTTTCCCACTTGCTTAAGTAAATAAACTGTTCCAGCAAATGCTGCTATTTTTAAAAATGAAGAT
ACAATTGTAGAGAGCTTGTTCCAAACCCATCCTACAGCACGGTAGACATGGGCGAGAG-
TAACTCTTTTCACAGTATGAACTATTGATAATATTCGCTCTCTGAGGTGTTCACGAGATAAAGCATACATGTGCTCATATTG
CCATTTAGCAGCTGAAACCATGTCACGCTGTTCTTCAGCAGAAAGCAATGAGAATAAATTT-
GCTGTATATGTAAATGACTGGTTTATTGAACAGAAAAATGCTATATCTAATGGTATATGATAAGTTTTTGAATATTATTTA
AATTTTTCTTAATAGCAATAGAATTAGTAATATGAAAATCAA-
GATCCGACACATCCATGTGCCAATCTCCGTCAACATAATGTAGACGGCGCAAAAATTCAATGGATATGCGAGAATTTCCTT
GTTCAATATGTTCATCGGAATATGGTTTACGATTAGGATAA-
TAAAATTTATGCTCTGAATTTATCTGTTTAAAGAAGGACATCATTTTTGGTTTGTCCCACATGAATCGCGTTTTATAATTTTG
ATAAGAGGACTCCATAACTTGATCTGATTTTAAATAATTATAAAAATTCTCTCCTGG-
GAAGGTTCCTGACTCGGAACAACCCCAGTTAGTATAGTAAGACCAAATCGGGATAGCTGGGTAAAGATTGCCTTCAGTAT
TATGCACTAATTTAACAACCATTTGTTCTTTCACTAAAGGATCAATAAC-
GCGTTTATTTAATTTTTGCATAATACGCTGACGCCTAGATGTTTCAGGTCCAGGATTTCCAGGGACAATATCTTGATATGTTT
CCTTAGGGAATGGATTTGGGGCCTTATTGTCTAAATATGAAAAATAACGATAATCTGAGA-
TAGTAAATTGCTCCTTCATAGCGCACTTATGCATTAATAATAAAACTAAATCGCGCTCTGGGCCTGTTAAATCATGAGATC
GAATATGCTCAGTTAGCTTTTCAAGCTTAATCTCTTCGTCTGTTAAAGTAAGACCCAAAA-
GCTCATTAGGATCCCCGTAACTAGACTCCCAGTCATCATCATCGATGGTGCCTGCTGTTGGCATTCCTTCAGTGGAATCCAA
AAATTCTTGTGTTATTCCATCGATATCAAGATCACACATATCTGGAAGTACTTGATCTAAC-
GTTTGAGCATAAACACGCTTTCCAAGGGGATCATCCATACCATATTGAAGGGAAGCATCCAAAAATTTATCTGAAATAAG
TTTAAATGTGGTGGAAGCAGCAACACCATCTGAAATGTCATCTGATTGCACAAAAA-
TATTATCAATTTCAGCGAAACATTCCATGACATGCTCGTACTTTTCTTTCACAGTGAGTTTTTGTTCACCTGATCTAAGATGT
TGATAACTAACTGCAACATTCTCAACAAAAGTATCAAATGTCCAGTTAGTGCATGGAACTGTCATTCCTTTCAAAGGACG
>TRINITY_DN4257_c0_g1_i2
TGATTTTCCAATACCAGGTTCCCCTACAAACTGTACATGAAACATTGCAGGTTTAAAATTT-
GAAGTAGATCGAATACGGTAAGTATAATTAGACAAAATTTTAACGTCATTCCAGTTACGATGGATAACCATTAATGATTCA
CGTGATATAAATTTGGGGTCTTTCGAGCAAGCTGTAAGAAAAGCAAGACCTTCTGACATAA-
TACGCTCCGCAACTTTCAAAAGTTCCTCTGAAACACGAATCGCGTTACGTCCAGTGTCAGTTGAGAAAAATTTAAGTTTTA
CAAGCCATCTTGCCACTGCTTTTTCATTGGTCATAGCTTGCTCAGTAATACCTAAAA-
TATGTTCTTGAATATATGAAGTTACTGATTTTGAAATAATAACAAAATATTTATAAATGCGGTCAATTCCGAACATGCCACT
TCCAACCCAATGGAAGCCTTTGCACATCCCAATAACCATGCCGGCAATTGTT-
GTATACTCATTAAAACGCATGGTTCCTGCATTACGAAGACCGCAAGTGTGTTTAAAAATCATAATTAACAATGCTG
>TRINITY_DN4506_c0_g1_i1
CCCGATTTTAATCCGTAAAACAGAGACCAACCAAACGGGAGCTATTCCTT-
GTGCCAGGCTTTCTGTTGAATAGTAAACATAAGCCTCCAGGCTATGGATCAATACCCAACCTTATTGCACATAATACATGT
CTAACTTTGCAATAAATGTGATACATCATCTCCAACTCTCAAAGATTTTCGAAGAT-
TTCTTTTCAAATCTAAGAGGTCCGGTTCGTCACCAACCTCACTCGCAAGATCTTCATCACGCAAAAGAGGTTTATAGTCAA
CAGTGTCATATGAACTTAAATCTCTAATAGTCCGTGGCCGTGATCGGTGTAATGGTGTATTT-
GTTTGAACTGGATCATGTGCTCGTGCAACTGCTTTCATAGTGTTTTCAAGTTGCGCAACTGCTTTAAAACCACCAAGCCCAT

CTGTTGTGTATGTTTTTGAGGGTACTTGGATAATATTTATTTTGCCATCAGCATATTTCGAA-
TAAGGAGTTAGAAAATGGGCATTGTAAGGTAAAAATGGATCTGCAAATTGAAAATCATCTCCCGCCCGATATTCAAGCTC
AAATGTAATCTGTGAATTTTGAACCGATGATTCCAATTTTCCACGAAGGCCAAACAAAA-
TATAATTGTCTCCATGAGGTTCATTTCGGATGTTACGCAAACGGTCATATTGAATAGGACCTTGACTTTTATTATAAGACAC
AAAATTTTTCGCAGGTCTTGTACGGTTCCCTATCCAATTAAATTGATCATAAAAAGGGACCG-
GATATTCAAAGGGAACTTCAACCTCAAAATGACGGAACATTGCGGTATCTCCCATTACATATGAATTTATCATAGATTCAC
GATAAGAGCGGTCCTCTCTCTGAATTCCAGGTGTTGTATTTTGAGTTGGATAAATACCAA-
TAATTGAGGGTGAATTACGAACTACAGATGAAAATATATAGCCTTGCGTGGTAAAGCCTGCCACAGTGCGAATTCTATAA
TGCATTGTTCCTCGCCATTTACGAAACATGGTGGAAAGAGAAGCCAAAA-
TAGGAGGCTGATCATATTGCGTTATATAAACAGCTTCATCATCAGGATTAATTTGTGTATAATTTTTACCTGCATGAATAAC
ATTTCGCATGTTGTTATAAGCGTAGGCTTCATATCCCACTAAAGTT-
GTTAAATGCCTTTTATCTAGCTCAGGAATCTTATCTGTATTATTCCAAGGCGCATCTGTTGATGAATCTTTATATTGGTAAAA
CAGTGTTGGAATAAAAGGCCCATTCCGGATTGCAAAAATATAATCTCGGTCATTTCCAG-
TAAAAGGCAAATTTAACGTTACTCTTATGCCCATTGGTTGCCATTGATGAATAAGATGATCAAAATCAAAAGGCC
>TRINITY_DN5829_c0_g1_i1
TGAAGCAACATTGATTCGC-
TACTCAACAAAAATTACAGGTCAATTATTTAATCCTCGTTTAATTTATTATTTAACATGGAAAAATGCAATTTTAGATCAAA
TGACACCTACAGAGCGTTATCGAGATTCATTACAGATGCCTTTGTCTCATCTACATGGA-
GAACCAACTATGTTTCGCATAAAAGATAACATTAATGAAGCTGTGGATGAGAATATCCCCAAAGTTCAATCAGCCATTCA
AGATTCTTTAAAAGAATTGTTGAAGTCTCCTGAAATTAAAGAATCTGTTACTAATTTAG-
TTACTGACTCCATGATCCCTACTCTTGAAAATTTTGAAAAATCATCTGAAAAAGTCAGTAATTCTGTATTAGATAACTTAAA
AACATCTATGGCACCGTTGATAGAGCAAACATTTTCCTTATTTTCAACCGTTAAC-
GGCCTTATAGATTTCATGAAATCTATGCTTAAACAGGCCATTGATGCTTTTCCTAAGGAGCTTTTTGGATCAAAGTTAAATC
TTGATATTTCTCCCGAAACCTTATTAAATCTTCTTAAGTATTA-
TATTATTTATGTTAATGTAGAGTCAAAGCCTTTAAAAATAGCTTTAATTTATCT
>TRINITY_DN9440_c0_g1_i1
AGACTTATCAGGAATTCGTTCAGAATGGTCAAGAATTGAAAAACAATCTGCG-
CAGAATGCGCTTAGTCAAACTACGCCAGCGATCCCGTCGACTTCATCCGCGCCCCAGCCTACAGTCCCTGTTGCCCCTTTG
GCGGCTGCAGAAACCAAAGGACCTGCAAAAATAGCTCAATCTAAAGATGTTGCAAAGGTT-
GCTAGCGCTGTTACAGGTGTTGGCAATGCAGCTTTGTCCGTAGCCTCCTTAACTGGACCTGCTGGCATCGCTGCTGCAATTA
ATGCTGCTGCTGGTGCTGCTACTGCTAATGCAATTGATGCAGGAAATAAATCAAC-
TATTGCTTCGGACTATGTTGCTAATACAAAAGTTCAAGGATCTCAATCAACTCACCAAGCGCAACTTTTGAAGGAGATTGA
TACTGCTCATGCTAATGTTACAGCCTCTGGAGCTCAAATAGGAGGCTTGTTTGGCCCTGTT-
GGTGCATGGTTGGGGAGTTCCCTTGCCAATGCGATTCAAGACTCATCTCCAAAAGACACTTACAATGACTTAAAAACAGG
ATATTCTTTTGAAGGCCGCTTTAATCCACAAGATACTGGATCTGTAAATTCAG-
GAACAACAGCTAATTTATCAGGTCAATCAAATATTCAATCTAATATATAATGTCATTAAATGAAGAAATTAATGCAGATA
CCCATAATCCTGTTCGAAATTATGGGTTGCCACAGAACCCTGTACCCCAAACAAA-
TACTGGGCTACTTGAGCATACAGGAATACCTTTTGAACCTCTTGAAAATATTACTTCTTTACCTCCACAGTTTTCATGGATG
GTAGAACAATATAAAGTCTTAACAACTTTGACAGTTAGTACAACTAACATGGTAGGATCAAC-
TATCTATTCTACAGATGTTCTACATCCACATAATAAAAATATTATTTTTCGATCTTACCCCAATTGGGAGAGGGTTCCTTTTG
CATCATCTATATGGTGGAAAGGAATTGTTTCCTATCGCCTGTCGATTATTAAAC-
CTCCTCGAGTTACTGGCAAGTTATTAGTTCGATACAGACAGGACAACTTTCTTAATTTTGACGGTTCGCATGATGACAACG

ACAATGTTATTGATAACACCTACCGTTCAATATTAAAGGAATGGGATTTATCGGAT-
TCTAATCATTTTACATTTGACATTTCAGCTTCTTTGCCAATTAGAGCTCGTCCTACTAAGTTCCCTGGACTTAAGAATGCTTC
ACCCTCTTCGGATTCTATTATTTATGCTTCCTCCATGACACCATGGATTGATTTTTCTATGG-
GAGCATTTACATTAGAAGTTGCTGAAACGCTAGCTCCAGGTAGTATATTTCCTGATACATATTCAATTATAGTGGAGAGAG
CTTTTAAAAATACTGAGTTTATGATTCCCACTGATTCTAAATCCAC-
TTATTCATTATGTGTCAAAGAATCACCATTTTATACCCAACCATGAGTAACGAAGAACCACAAGTCACACCAGAACAAAA
TGTTCAATTGCCCAGCGAGTCGCATAAACTTCCTGAGCAATCTAGAATTCCTCAATGTAG-
TATACCTAGGCCTTTTGATTTTGATCATCTTATTCATCAATGGCAACCAATGGGC
>TRINITY_DN9482_c0_g1_i1
CTTGCCAATGCAATCTAATATGCTTAATATACCATCTGCCCCTGGAACTGCTTTTGATGATA-
GATCATCAGTCGTGGCTACTAAGTGGGCTTTTGAGCAATTAGTATCTCAGAAACAACTTATGACGACTATTACAATTCCCA
CAAATGGAACTGGAACTACCCCTTTATTTCTATTTCAAAATTCATGGAATAA-
TATTCAGAAATTGCATTTTAGAAATTTAGCAAATTTGTTTTTCTTAAAATCATGGAAATGGCATTTAACATTTGAATTCCGG
TCTAATTTCCAAGAAGTTGGAATGATGACTATCGCGTATGCCAATGTACCCTT-
GGATGCCGTCCCTTATTTAACTTCATCTCCTATTTCTTATGTCGATACCCAAGTTACTAAAAATGACACCCCCGGTCAGAAT
GGGCTAATTACAGCAGCTAATATAGTTGACCATTCTCTTTTTAATTTAA-
TAACAATGAATCAATTACCACATGTTCATGTAATGCTGGGAGAGAATCAGGATGTTGAGTGTACATTTGACTGGCTATCAC
CTTTTAAATCATCTTATACTAAAGTCCAGCCTTTTAAGCCTAGTTATGTTTTTGGCGA-
TAAACTTGAAGACCCAAATGACCCTTATTATGATATGGGATTTGTTTATTTAGTTAATACTATTCCTTTGACAACTGCATCC
GGTGTTACACCACATGCCACAGTGAGAATCTGGTCGCATTTAACCGATGTTGAG-
TATTCTGGCTATGTTCCTCAAGACAGGATAATATGAATTTTGTTAAATCATCAAATTCTATTTCATTATCTGGCCCTAATGTT
TCCTGGACTACAAATCCAATTTACCAATCTCCC
